# Microbiota modulate immune cell populations and drive dynamic structural changes in gut-associated lymphoid tissue

**DOI:** 10.1080/19490976.2025.2543908

**Published:** 2025-08-13

**Authors:** Pačes Jan, Malinská Nikola, Tušková Liliana, Knížková Karolina, Grobárová Valéria, Zadražil Zdeněk, Hudcovic Tomáš, Michl Anna, Šrůtková Dagmar, Schwarzer Martin, Boes Marianne, Černý Jan

**Affiliations:** a Laboratory of Cell Immunology, Department of Cell Biology, Faculty of Science, Charles University, Prague, Czech Republic; b Laboratory of Gnotobiology, Institute of Microbiology of the Czech Academy of Sciences, Nový Hrádek, Czech Republic; c Center for Translational Immunology, University Medical Center Utrecht, Utrecht, The Netherlands

**Keywords:** Phenotypic plasticity, Microbiota-induced immunity, Gut-associated lymphoid tissue (GALT), MHCII-EGFP knock-in mice, Germ-free and gnotobiotic models, Lymphoid tissue morphogenesis

## Abstract

Inbred mouse strains provide phenotypic homogeneity between individual mice. However, stochastic morphogenetic events combined with epigenetic changes due to exposure to environmental factors and ontogenic experience result in variability among mice with virtually identical genotypes, reducing the reproducibility of experimental mouse models. Here we used microscopic and cytometric techniques to identify individual patterns in gut-associated lymphoid tissue (GALT) that are induced by exposure to microbiota. By comparing germ-free (GF), conventional (CV) and gnotobiotic mice colonized with a defined minimal mouse microbiota (oMM12) MHC II-EGFP knock-in mice we quantified antigen-presenting cells (APCs) in the lamina propria, cryptopatches (CP), isolated lymphoid follicles (ILFs), Peyer’s patches (PPs) and specific sections of the mesenteric lymphoid complex. We found that GF mice had a significantly larger outer intestinal surface area compared to CV and oMM12-colonized mice, which partially compensated for their lower density of the villi in the distal ileum. GF mice also contained fewer APCs than oMM12 mice in the Iamina propria of the villi and had a significantly smaller volume of the solitary intestinal lymphoid tissue (SILT). In both GF and oMM12 mice, PP follicles were significantly smaller compared to CV mice, although number was similar. Concomitantly, the number of pDCs in PPs was significantly lower in GF mice than in CV mice. Moreover, the cecal patch was dispersed into small units in GF mice whereas it was compact in CV mice. Taken together, we here provide further evidence that microbiota regulates SILT differentiation, the size and morphology of PPs, the cellular composition of mesenteric lymph nodes (MLNs) and the morphology of cecal patch. As such, microbiota directly affect not only the functional configuration of the immune system but also the differentiation of lymphoid structures. These findings highlight how standardized microbiota, such as oMM12, can promote reproducibility in animal studies by enabling microbiologically controlled experiments across laboratories.

## Introduction

Microbiota maintain gut homeostasis and modulate the function of other organs through a vast network of bi- or multidirectional connections known as the gut-organ axis.^1^ These microbiota-mediated axes include connections to adipose tissue, bone, heart, kidney, liver, skin, and brain, but the functional implications of these axes for immune homeostasis remain incompletely understood and are not the focus of this study. The gut-brain axis, in particular, encompasses not only neuronal but also hormonal, metabolic and, above all, immunological interactions.^2^

Highlighting the intricate interplay between microbiota and the immune system, mice show systemically decreased T helper (Th) cell proliferation and counts, along with locally reduced T (Treg) regulatory cell counts in PPs and MLNs after long-term antibiotic treatment.^3^ And while anti-inflammatory microbial metabolites predominantly derive from obligate anaerobic bacterial strains,^4^ a pro-inflammatory state can be caused by dysbiosis of gut microbiota. This condition is characterized by an elevated relative or absolute abundance of pathogenic bacteria, decreased commensal microbiota, and alterations in bacterial metabolism or spatial distribution in the gut.^5^ In numbers matching host cell counts, complex gut microbiota directly interact with the intestinal epithelial layer, maintaining epithelial barrier function, promoting antimicrobial peptide and immunoglobulin class A (IgA) production, providing protection against colitis, modulating innate lymphoid cell function, and inducing Th and Treg cell differentiation and proliferation in gnotobiological models.^6–9^

Yet, despite their genetic uniformity, these inbred mouse strains, commonly used in immunological research to ensure phenotypic consistency, also display individual variability. This variability arises from stochastic morphogenetic events, epigenetic influences, and differences in antigenic and cognitive experiences, indicating that genetically identical individuals can show phenotypic differences. Such heterogeneity is often overlooked in experimental design and data interpretation. Assuming that the microbiome may contribute to such phenotypic diversity, we chose the gastrointestinal tract (GIT) as a model organ due to its structured complexity, spatial organization, and accessibility for comparative immune analysis.

Gnotobiological models, including germ-free animals, provide an efficient and straightforward methodology to explore the interactions between the microbiota and the host. Using this approach, numerous morphological, numerical, and functional differences have been identified between specific pathogen-free (SPF) and GF mice, including lower B and T cell counts in the small intestine, decreased colonic Th cell counts, and a general reduction in memory and effector T cells and in regulatory Tregs.^3,10^ Various myeloid cell types, such as macrophages, neutrophils, and monocytes, are also decreased in GF mice.^10–12^ Beyond these differences, GF mice display differences in individual cell populations from SPF mice.

Gnotobiotic models are thus a powerful tool to functionally dissect microbiota-host interactions. In this context, GF, conventional (CV), and selectively colonized mice provide defined systems to examine how microbial complexity affects immune system development, particularly in mucosal compartments such as the GALT. The absence of microbial stimuli during early development leads to significant changes in intestinal architecture, such as a marked reduction in villi size and the number of ILFs.^3,13,14^ However, much of the literature describing morphological differences between GF and SPF animals, particularly regarding intestinal wall thickening or variations in the size of PPs and MLNs, is either outdated or based on studies conducted in rat or pig models.^15–18^ And while *B. fragilis* and *L. plantarum* monocolonisations can partly restore normal physiological and immune functions in GF mice, monocolonisation experiments do not fully replicate the original state. These findings highlight the need for diverse microbial communities for normal development and immune function and underscore the complexity of host–microbiome interactions.^19–21^

Notwithstanding efforts to reduce microbiome complexity, “mouse facility-specific microbiomes” significantly complicate comparisons of experimental results across institutions. Standardizing microbiome compositions for reliable comparisons may require defining a bacterial subset that reduces microbial alpha diversity while preserving the variability of major bacterial phyla. For instance, colonization with oMM12 brings the immune response closer to the physiological state.^22^ However, additional bacterial phyla must be introduced to fully restore the immune function and to match that of SPF mice.^23^ Nevertheless, oMM12 mice show no significant abnormalities in the development and quantitative characteristics of immune tissues or any significant difference in IgA plasma cell counts, total CD4^+^ T cell counts, Treg cell numbers, and Th17 cell numbers from SPF mice.^24^

The hierarchical composition of GALT includes multifocal structures such as PPs and numerous ILFs distributed throughout the intestine, in addition to the lamina propria, which fills the space between the intestinal epithelium and supportive tissues, and MLNs. These structures are in close contact with the microbiome, which prevents colonization by pathogens, facilitates chemical transformations and digestion, and produces bioactive secondary metabolites. However, our current knowledge of the variation of GALT diversity and function as a function of the microbiome primarily relies on qualitative data. As a result, the relevance of many findings remains unclear.

MHC II-EGFP knock-in mice allow precise localization of MHC II^+^ antigen-presenting cells in mucosal tissues. This model enabled us to visualize lymphoid structures *in vivo* and *in situ*, including major lymphoid organs and scattered GALT components, and to quantitatively assess their cellular and spatial characteristics under varying microbial conditions. This model has proved highly valuable for quantitatively characterizing the immune system at cellular, tissue, and whole-organism scales^25^ and for accurately identifying and counting major (lymph nodes) and minor (scattered mucosal lymphoid tissue) lymphoid organs.^26^ This model also facilitates a precise dissection for further analysis or cell/organ cultivation and enables label-free 3D histology through light sheet microscopy (LSFM), providing a noninvasive approach to studying tissue complexity while preserving cellular assemblies in their physiological context. As a control to GALT, corneal immune cell populations were selected, as another microbiologically challenged body interface, distant from the alimentary canal.

To evaluate the relationship between microbial complexity and GALT organization, we employed a quantitative comparative analysis using GF, oMM12, and CV MHC II-EGFP mice. This allowed us to assess microbiota-dependent phenotypic variation both between and within defined experimental groups. The analysis, which focused on characterizing not only the differences between the experimental conditions but also on capturing phenotypic plasticity within the experimental groups in terms of microbiome content, revealed that the oMM12 is more similar to the GF mice in some traits – a fact that needs to be taken into account when designing future defined “oligo” microbiomes.

## Materials and methods

### Animals

Conventional (CV) MHC II-EGFP knock-in mice^22^ on a C57BL/6 background were housed at The Center for Experimental Biomodels, First Faculty of Medicine, Charles University, under a controlled 12-hour light/dark cycle, at 22°C and 55% relative humidity, and provided with *ad libitum* access to a commercial grain-based ST1 diet (Velaz). Germ-free (GF) and OligoMM12-colonized (oMM12) MHC II-EGFP knock-in mice were housed and bred under sterile conditions in Trexler-type isolators at the Laboratory of Gnotobiology, Institute of Microbiology of the Czech Academy of Sciences, Nový Hrádek. The axenic status of the mice GF mice was regularly monitored by aerobic and anaerobic cultivation of fecal samples and swabs collected from the isolators and by gram staining of fecal smears.^19^ Mice were maintained under standard environmental conditions, including a 12-hour light/dark cycle, 22°C temperature and 55% relative humidity, provided with *ad libitum* access to autoclaved tap water, and an irradiated (50 kGy) mouse breeding grain-based diet V1124–300 (Ssniff Spezialdiäten GmbH, Germany). 8–10-week-old mice were euthanized by cervical dislocation. All experimental procedures were conducted in compliance with guidelines and approved protocols of the Czech Animal Care and Use Committee and the Animal Research Ethics Committee of Charles University. The number of experimental animals per group was typically 5–6 for morphological analyses, 4–9 for cytometric data, and 9 and 10, respectively, for identification poppulations in the cornea.

### Establishment of gnotobiotic OligoMM12 mouse colony

GF MHC II-EGFP knock-in mice housed in an isolator were colonized by 12 bacterial strains according to protocol described in.^27^ Briefly, the frozen OligoMM12 mixtures were purchased from DSZM (German Collection of Microorganisms and Cell Cultures GmbH), surface sterilized in 3% Virkon S and transferred into the isolators. The thawed mixture was used within 30 minutes after thawing. 8-week-old breeder GF mice were inoculated by gavage and intrarectally (50 uL each) twice within 72 h. The colonization was checked regularly by qPCR^27^ and by 16S rRNA sequencing. Two bacterial strains were bellow detection limits of either method, as previously described by others.^27–29^ oMM12 mice used in this study were 5–6 generation of gnotobiotic mice after initial colonization of the breeders.

### Sample collection and DNA extraction

Samples from the ileum, jejunum, and cecum contents were collected from three 8-week-old female C57BL/6 CV mice and from three 5-month-old C57BL/6 females colonized with the oMM12 bacterial consortium. The small intestine was sterilely removed and jejunal content was sampled from the middle part; ileal content was sampled from part 3 cm away from cecum. Samples were immediately frozen in sterile eppendorf tubes at − 80°C until processing. Total DNA was extracted using the DNeasy PowerSoil Pro Kit (Qiagen, Hilden, Germany; LOT181011328). PCR amplification negative controls and sequencing positive controls (mock communities; ZymoBIOMICS Microbial Community Standard, Zymo Research, USA; linear) were included and processed alongside the samples.

### PCR amplification, sequencing, and data analysis

Library preparation was performed by SEQme s.r.o. (Dobříš, Czech Republic) using a two-step PCR protocol based on NEXTERA technology, incorporating IDT® for Illumina NEXTERA DNA Unique Dual Indexes. The V4 region of the 16S rRNA gene was amplified using the primer pair 515F and 806 R. Library quality control (QC) was conducted using the Agilent Bioanalyzer 2100 with the High Sensitivity DNA Kit. Library quantification was performed using the Invitrogen Collibri Library Quantification Kit and the Quant-iT™ 1X dsDNA High-Sensitivity Assay Kit on a Qubit fluorometer. Following QC and quantification, libraries were pooled in equimolar concentrations. Sequencing was carried out on an Illumina NovaSeq 6000 platform using a paired-end 2 × 250 bp configuration. Raw sequence data quality was assessed using MultiQC (version 1.14). Paired-end reads were imported into QIIME2 (version 2024.2), where primers were trimmed and ASVs were generated using the DADA2 plugin. Samples from oMM12 and CV mice were processed separately. For the oMM12 samples, low-abundance features (minimum frequency < 40) were removed, and a rarefaction threshold of 16,827 reads was applied based on inspection of the rarefaction curve. Taxonomic classification of ASVs was performed using a pre-trained Naive Bayes classifier (scikit-learn) trained on the V4 region of 16S rRNA gene sequences from the 12 bacterial strains comprising the oMM12 consortium. A barplot displaying taxa per group was generated using the ‘mean-ceiling’ parameter to represent average relative abundances. For CV samples, low-abundance features (minimum frequency < 40) were removed, and a rarefaction threshold of 44,557 reads was applied based on inspection of the rarefaction curve. Taxonomic assignment was conducted using the VSEARCH classifier with the SILVA 138 database. Features with a relative abundance < 1% in fewer than three samples were filtered out using the qiime feature-table filter-features-conditionally plugin. A barplot of group-wise taxa composition was again generated using the ‘mean-ceiling’ parameter to show average relative abundances. Beta-diversity analysis was done based on weighted UniFrac distance matrices, which were obtained from QIIME2 and visualized in R using the qiime2R package (version 0.99.6). Principal Coordinate Analysis (PCoA) was performed, with PC1 and PC2 representing the major axes of variability. Clustering patterns are visualized as centroided individual samples, grouped by intestinal part. Mock community samples served as positive controls and were processed separately.

### Fluorescent stereomicroscopy

Mouse organs were dissected under a Zeiss SteREO Lumar.V12 stereo microscope for *in situ* imaging of the cornea and MLNs. The intestine was initially dissected and then imaged as a whole or sectioned and washed for villi quantification. For surface area measurements, five (*n* = 5) images of the entire small intestine were acquired at 9.6x magnification and subsequently stitched and analyzed using Arivis Vision4D software. To prevent stretching and gravitational deformation, the entire small intestine was immersed in PBS and then photographed in a relaxed position. The approximate value of surface area was calculated by first measuring the width (2 ×r). The mean radius (r) was determined by dividing the intestine into 5 segments of equal length, randomly selecting a measurement site in each segment, and averaging the 5 values. The surface area was then computed using the formula 2π x r ×v, where v represents the measured length. For villi quantification and PP analysis, intestinal sections were imaged at 16x magnification. Villi were quantified in 3 randomly selected 1-mm^2^ squares in both areas (proximal jejunum approximately 5 cm from the start of the small intestine and distal ileum approximately 2 cm from the end of the small intestine) selected in each of the 5 mice. Individual villi were manually segmented using Arivis Vision4D software. In MHC II-EGFP knock-in mice, the overall fluorescent signal of the small intestine surface reflects the abundance of the APCs localized in the 3D structure of individual villi. As a result, the signal is not homogeneous, which enables a precise quantitation of villi.

### Light sheet fluorescence microscopy (LSFM)

In the cornea and individual villi, cells were counted by LSFM. To quantify corneal cells, eyes were washed with PBS and visualized under a Zeiss Z.1 light sheet microscope equipped with a 1.38 refractive index (RI) chamber. Each sample (*n* = 11) was scanned from the limbus to the center of the cornea.^26^ To quantify cells in individual villi, two approximately 6-mm-long segments were excised from each mouse (*n* = 5, or 6 for CV mice), one from the proximal jejunum (in the area of the first PP) and the other from the distal ileum (in the area of the last PP). In each segment, 3 randomly selected views were imaged, and cells were counted on one villus from each view. Intestinal segments were fixed in 3.8% paraformaldehyde at 4°C overnight and then cleared using a modified Clear Unobstructed Brain Imaging Cocktails and Computational (CUBIC) analysis protocol for 7 days (35 wt% dH2O, 25 wt% Urea, 25 wt% N,N,N‘,N‘-Tetrakis(2-Hydroxypropyl)ethylenediamine (4NTEA), 15 wt% TritonX-100).^26^ The cleared samples were rinsed 3 times, for 1 hour each time, in CUBIC wash solution (0.5% BSA, 0.01% sodium azide, 0.01% TritonX-100 in PBS) and then stained with DRAQ5™ Fluorescent Probe Solution (Thermo Scientific) at a 10000x dilution in PBS with 0.5% BSA and 0.01% sodium azide for 7 days. Following nuclear staining, the samples were washed again and kept in CUBIC 2 clearing solution (23.4 wt% dH2O, 22.5 wt% Urea, 9 wt% triethanolamine (TEA), 45 wt% Sucrose, 0.1% (v/v) TritonX-100) for 4 days for refractive index matching. The samples were visualized under a Zeiss Z.1 light sheet microscope with a 1.45 RI clearing chamber.^26^ LSFM data were analyzed using Arivis Vision4D software.

### Flow cytometry

Mice were euthanized by cervical dislocation, and specific organs were excised under a Zeiss SteREO Lumar.V12 stereo microscope. The MLN complex was rinsed in PBS. After removing fat and connective tissue, individual MLNs were separated, and the excised organs were then cut into small pieces using scissors in RPMI 1640 medium with 3% fetal bovine serum (FBS). The fragmented tissue was digested in collagenase (0.1 mg/ml) for 40 min at 37°C. Enzyme activity was stopped by adding 5% FBS and 2 mM EDTA. Cell suspensions were filtered using a 40 μm cell strainer, washed in PBS, and then resuspended in anti-mouse CD16/32 to block Fc receptors. For staining, blocked samples were treated with a myeloid or lymphoid panel along with True-Stain Monocyte Blocker (Biolegend) for 25 minutes at 4°C. Cell viability was assessed using SYTOX™ Blue Dead Cell Stain (Invitrogen). The samples were measured on BD LSRII and Cytek Aurora cytometers and subsequently analyzed with FlowJo 10.7.1 software. The myeloid panel included the following markers: Ly6G/BV421, Bst2/BV605, F4/80/PE-Dazzle 594, CD19/APC, CD11b/PerCP-Cy5.5, Ly6C/BV711, CD45/A-700, CD11c/PC7, MHC II/APC-Cy7 (Table SI). The lymphoid panel included the following markers: CD3 PerCP/Cyanine5.5, CD4 PE, CD8 PE/Dazzle 594, CD25 PE/Cy7, NK 1.1 Brilliant Violet 785, γδ TCR APC, TER-119 Pacific Blue, CD19 APC/Cyanine7 (Table SII). Because a transgenic MHC II-EGFP mouse model was used, all MHC II-expressing cells were also detectable in the GFP channel.

### Immunohistochemistry

Small intestine segments were fixed in 3.8% paraformaldehyde in PBS at 4°C in the dark. The following day, the segments were washed three times in PBS, transferred to 30% sucrose in PBS, and then stored at 4°C, in the dark, overnight. Subsequently, the tissues were embedded in mounting medium Cryomount on dry ice and stored at −80°C. Using a Leica CM1860 UV cryostat, 10-μm-thick cryosections were prepared on Epredia’s Superfrost Plus Adhesion Microscope Slides and stored at −80°C. Before staining, the cryosections were permeabilized with 0.5% Triton X-100 in PBS, washed three times with 1% PBS, blocked with 1% BSA in PBS for 20 min, and then washed twice with 1% PBS. Lastly, the nuclei were stained, and the samples were mounted using Fluoroshield with DAPI (Sigma-Aldrich) for visualization under a fully automated ZEISS AxioScan Z1 microscope with a 20x objective. Data were processed using ZEISS Zen 3.7 software and FIJI software.

### Confocal microscopy

The small intestine segments were prepared as described above for immunohistochemistry, and 20-μm-thick cryosections were prepared using a Leica CM1860 UV cryostat and stored at −80°C. The resulting cryosections were permeabilized with 0.5% Triton X-100 in PBS and then washed three times with 1% PBS. The samples were mounted using Fluoroshield with DAPI (Sigma-Aldrich) to stain the nuclei and visualized under a ZEISS LSM900 confocal microscope with an Airyscan with a 63x objective. Data were processed using ZEISS Zen 3.7 software and FIJI software.

For imaging of SILT, 20–30 µm wholemount sections of small intestine were stained with anti-CD4 (GK1.5), CD19 (6D5), F4/80 (BM8), and CD45 (30-F11) antibodies. Confocal microscopy was performed using a Zeiss LSM880, and signal intensity and co-localization were analyzed using Arivis 4D.

### Statistical analysis

Statistical analysis was performed using GraphPad Prism 10.2.2 software. For pairwise comparisons, the unpaired t-test was used when both datasets showed normal distribution as determined by the Shapiro – Wilk test. If the Shapiro – Wilk test indicated non-normal distribution in either dataset, the non-parametric Mann – Whitney test was applied.

For comparisons involving more than two groups, one-way analysis of variance (ANOVA) with Tukey’s multiple comparison post-hoc test was used when all datasets met the assumption of normality. When the Shapiro – Wilk test revealed non-normal distribution in any of the groups, the non-parametric Kruskal – Wallis test was employed. The probability levels were denoted as follows: *p < 0.05, **p < 0.01, ***p < 0.001, and ****p < 0.0001. The results are expressed as mean ± standard deviation (SD).

## Results

### GF mice have a larger outer intestinal surface area but lower density of villi and lower density of APCs than CV mice

16S rRNA sequencing analysis confirmed the colonization by 10 bacterial strains in oMM12 gnotobiotic mice. *Bifidobacterium longum* subsp. *animalis* YL2 and *Acutalibacter muris* KB18 were not detected in jejunum, ileum or cecal content of colonized mice, a phenomenon previously described by others^27–29^ (Fig S1A,B). In CV mice, the small intestine was dominated by Lactobacillus and Muribaculacea, with bacterial diversity increasing along the GIT from jejunum to cecum. In cecal content mouse opportunist pathogen Helicobacter spp. was detected (Fig S1C,D).

**Figure 1.**
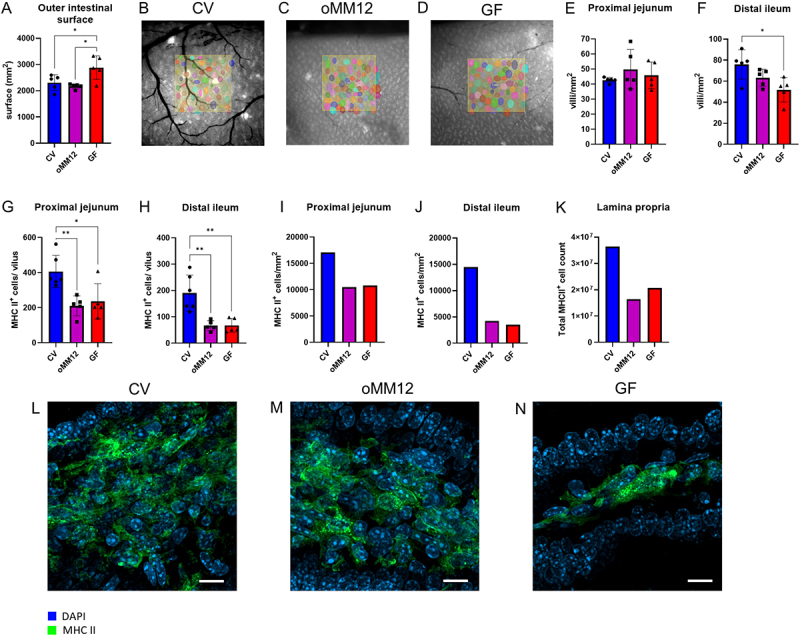
The intestinal microbiota affects the development of the lamina propria at the morphological level and in terms of the representation of antigen-presenting cells (APCs) and the localization of MHC II: (A) In each mouse model, i.e., CV, oMM12 and GF, the external surface of the small intestine was measured in five mice. The difference between GF and both other models (CV and oMM12) is statistically significant at *p* < 0.05, as determined by one-way ANOVA followed by Tukey’s post-hoc test. Illustrative images of individual 1-mm^2^ regions of the distal ileum of (B) CV, (C) oMM12 and (D) GF mice were used to calculate the average number of villi per 1 mm^2^ of the outer surface. All images were acquired under a fluorescent stereomicroscope at 16 x magnification. The density of villi (villi/mm^2^) was measured (E) in the proximal jejunum and (F) in the distal ileum. The height of the column represents the mean, the error bars display the standard deviation (SD), and the individual points indicate the measured values. The difference between CV and GF in the distal ileum was statistically significant at *p* < 0.05, as determined by one-way ANOVA followed by Tukey’s post-hoc test. In both the proximal jejunum (G) and distal ileum (H), GF and oMM12 mice exhibited significantly reduced MHC II^+^ cell counts compared to CV conditions. In the proximal jejunum, the comparisons GF vs. CV (*p* < 0.05) and CV vs. oMM12 (*p* < 0.01) were both statistically significant (*p* < 0.01). In the distal ileum, GF vs. CV and CV vs. oMM12 was significant at *p* < 0.01. All analyses were performed using one-way ANOVA followed by Tukey’s post-hoc test. These APC counts per villus are mirrored by the calculated values of APC density in lamina propria of the (I) proximal jejunum and (J) distal ileum and (K) by the total APC count in lamina propria, calculated from the APC counts per 1mm^2^ and total outer surface area of the intestine. Each column corresponds to a specific condition, i.e., CV, oMM12 and GF mouse models. The height of a column represents the mean, the error bars display the standard deviation, and the individual points indicate the mean values per individual mouse (*n* = 5 in GF and oMM12, or 6 in CV model). Significant differences between models are marked with an asterisk (*p < 0.05; **p < 0.01), according to ANOVA with Tukey’s multiple comparison post-hoc test. Confocal microscopy images highlight differences in the subcellular localization of MHC II (green) in the lamina propria of the small intestine of (L) CV, (M) oMM12 and (N) GF mice, with nuclei stained with DAPI (blue). Cryosections of the small intestine were observed under an LSM900 confocal microscope with Airyscan using a 63x objective. Scale bar: 10 μm.

As shown in Figure 1A, the outer surface area of the small intestine was significantly larger in GF mice (2882 ± 451 mm^2^) than in CV (2310 ± 303 mm^2^) and oMM12 (2170 ± 94 mm^2^) mice (*p* < 0.05, one-way ANOVA with Tukey’s post-hoc test), indicating a higher volume. However, the individual values of surface area showed a significantly higher variability in GF mice. Representative examples of the intestinal morphology are provided in Figure S5.

We chose proximal jejunum due to its minor bacterial colonization and distal ileum with a high bacterial load. We quantified the intestinal villi along the different segments of the intestine (Figure 1B-D). In the proximal jejunum, villi density was similar among all mice (42 ± 2 villi/1 mm^2^ in CV, 50 ± 13 villi/1 mm^2^ in oMM12, and 46 ± 9 villi/1 mm^2^ in GF mice; *p* > 0.05, one-way ANOVA) Figure 1E. In the distal ileum, the density of villi was significantly lower in GF (52 ± 12 villi/1 mm^2^) than in CV (76 ± 13 villi/1 mm^2^) mice (*p* < 0.05, one-way ANOVA with Tukey’s post-hoc test), with an intermediate value in oMM12 mice (63 ± 8 villi/1 mm^2^) (Figure 1F).

Differences in the abundance of APCs between CV, GF, and oMM12 mice were assessed by LSFM. For all conditions, MHC II^+^ cells were manually counted in three representative villi from each mouse to calculate the average value per mouse in the proximal jejunum and distal ileum. In the proximal jejunum, the mean number of MHC II^+^ cells per villus was significantly lower in gnotobiotic (211 ± 57 and 236 ± 101 in oMM12 and GF, respectively) than in CV (406 ± 92) mice (Figure 1G) (*p* < 0.01 and *p* < 0.05 one-way ANOVA with Tukey’s post-hoc test). In each villus of the distal ileum, MHC II^+^ cell counts, on average, were also much lower in oMM12 and GF (67 ± 19 and 68 ± 26 cells, respectively) than in CV (190 ± 69 cells) mice (Figure 1H) (*p* < 0.01, one-way ANOVA with Tukey’s post-hoc test).

Using the density of villi and abundance of MHC II^+^ cells in individual villi, we calculated the overall density of APCs in small intestine regions, which mirrored the aforementioned patterns. In the proximal jejunum, the mean density of APCs was higher in CV (17,059, cells/mm^2^) than in oMM12 and GF (10,508 and 10,809 cells/mm^2^, respectively) mice (Figure 1L). In the distal ileum, the mean density of APCs was also higher in CV (14,57 cells/mm^2^) than in oMM12 and GF (4,218 and 3,515 cells/mm^2^, respectively) mice (Figure 1J). Combined, these surprising results demonstrate that oMM12 and GF mice contain similar APC counts, albeit less so in the distal ileum than in the proximal jejunum because of bacterial loads. In summary, GF mice exhibited a larger intestinal surface area but lower villi and APC density compared to CV mice. This highlights the essential role of a complex microbiota in shaping intestinal morphology and immune cell distribution, as the oMM12 model failed to fully restore a normalized phenotype.

From the data above, we estimated the total APC counts in the lamina propria. APCs in villi accounted for approximately 80% of their total number (data not shown), so the data shown in Figure 1K underestimates the total counts. Nevertheless, the total number of MHC II^+^ cells in the lamina propria was approximately 2-fold higher in CV than in GF and oMM12 (approximately 36 vs. 20 and 16 million cells, respectively) mice. The oMM12 mice displayed the lowest total count of small intestine APCs, mostly reflecting their almost normal, non-expanded intestinal surface. In GF mice, the lower density of villi, with fewer cells, was only partly compensated for by the much larger intestinal surface area.

These mice differed not only in the abundance of APCs in villi but also in the intracellular/plasma membrane expression of MHC II. In CV mice (Figure 1L), the villi contained many APCs, with MHC II expression almost exclusively localized in the plasma membrane. In GF mice (Figure 1N), by contrast, MHC II expression was mostly intracellular (green dots in cells). oMM12 mice had intermediate distribution of the intracellular vs. plasma membrane MHC II expression in villi (Figure 1M).

### GF mice display a significantly smaller SILT count, area and volume

**Figure 2.**
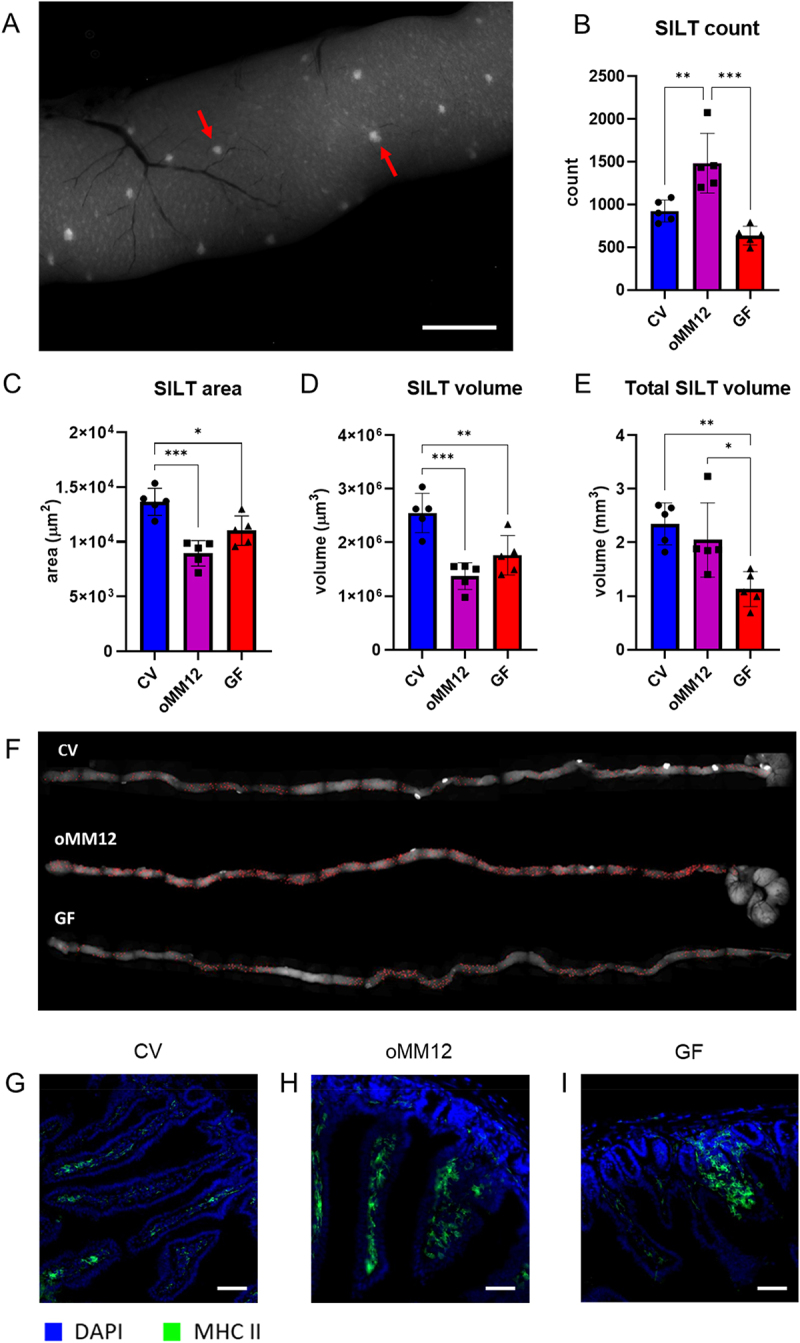
Microbiota affect SILT differentiation: (A) Fluorescent stereomicroscopy image of the proximal jejunum of an oMM12 mouse; red arrows indicate individual SILT follicles (scale bar: 1 mm). (B) Total numbers of SILT follicles in each model. oMM12 mice exhibited significantly more SILT units than both GF (*p* < 0.001) and CV (*p* < 0.01) mice. (C) Mean area of individual SILT follicles measured from fused stereomicroscopic images of the whole intestine. CV mice showed a significantly larger SILT area compared to GF (*p* < 0.05) and oMM12 (*p* < 0.001). (D) Approximate volume of each SILT follicle calculated from 2D data. GF and oMM12 mice displayed significantly reduced SILT volume compared to CV (*p* < 0.01) and (*p* < 0.001). (E) Total volume of the SILT compartment, estimated by multiplying the total number of follicles by the mean volume per structure. The bars represent the mean ± SD for each model. Significant differences were observed between GF and both CV (*p* < 0.01) and oMM12 (*p* < 0.05) mice. Significant differences between CV, oMM12, and GF mice are marked with asterisks (*p < 0.05, **p < 0.01, ***p < 0.001, one-way ANOVA with Tukey’s post-hoc test). *n* = 5 for GF and oMM12 mice; *n* = 6 for CV mice. (F) Representative images of the distribution of SILT in the small intestine of CV, oMM12 and GF mice, showing SILT units in red. PPs are visible as distinct white spots. SILT follicles were identified and manually counted from stitched images acquired with a Zeiss SteREO Lumar.V12 stereomicroscope at 9,6× magnification. Image stitching was performed using Arivis Vision4D software. (G) Normal villi in an CV mouse, in contrast to thickened villi containing an increased number of APCs in (H) oMM12 and (I) GF mice. Cryosections of the midsection of the small intestine were observed under a Zeiss Axioscan Z1 microscope with a 20x objective. Scale bar: 50 μm.

In the intestinal lymphoid tissue, the lamina propria is followed by CPs and ILFs. Both CPs and ILFs contribute to the SILT and, as with intestinal villi, can be easily identified and measured in MHC II-EGFP knock-in mice. To determine the total number of follicles, we counted individual follicles on one side of the small intestine using composite images acquired under a fluorescent stereo microscope (Figure 2A) and multiplied the final number by two (Figure 2B). The average number of SILT units was higher in oMM12 (1484 ± 349) than in CV (924 ± 128; *p* < 0.01, one-way ANOVA with Tukey’s post-hoc test) and GF (637 ± 109) mice (*p* < 0.001, one-way ANOVA with Tukey’s post-hoc test). Considering their markedly reduced APC counts in the lamina propria (Figure 1K), oMM12 mice likely develop a compensatory mechanism for immunological competence.

Although CV villi were clearly distinguishable from SILT units, intermediate structures were identified in oMM12 and GF mice, which we have named immunovilli. Consisting of thickened villi, these immunovilli tend to contain many MHC II^+^ cells, especially at their base. They are narrower than SILT units, albeit slightly widening as they extend toward the lumen, where they form villi. Exclusively found in GF and oMM12 mice, these intermediate villi may be counted as villi or SILT units in the stereomicroscopic quantitative analysis, depending on their size (Figure 2G,H,I).

While manually counting individual SILT units, we encircled fluorescent signals in the small intestine using a measuring tool to quantify the area and volume of each SILT unit. CV mice had the largest CPs and ILFs (13.656 ± 1.244 μm^2^ surface area and 2.552.017 ± 367.988 μm^3^ volume, with 2.348 ± 0.3914 mm^3^ total volume, calculated as SILT volume ×SILT count), suggesting that these structures were physiologically functional and morphologically differentiated in this mouse model. oMM12 mice had the highest SILT counts but the smallest SILT units in area (8.940 ± 1.159 μm^2^) and volume (1.372.631 ± 249.109 μm^3^), on average, resulting in the compensation of the total SILT volume (2.047 ± 0.693 mm^3^). GF mice had a markedly reduced SILT compartment, with a 11.019 ± 1.348 μm^2^ mean area, 1.760.852 ± 368.552 μm^3^ mean volume and 1.131 ± 0.325 mm^3^ total volume (Figure 2C–E). Both volume and surface area were significantly larger in CV mice compared to oMM12 (*p* < 0.001, one-way ANOVA with Tukey’s post-hoc test) and GF mice (*p* < 0.01 and *p* < 0.05, one-way ANOVA with Tukey’s post-hoc test). Total volume was significantly greater in CV (*p* < 0.01, one-way ANOVA with Tukey’s post-hoc test) and oMM12 (*p* < 0.05, one-way ANOVA with Tukey’s post-hoc test) mice compared to GF mice.

Highlighting the role of microbiota in the differentiation of GALT structures, both oMM12 mice and GF showed an irregular SILT distribution, in contrast to the even distribution of this tissue in CV mice. Representative datasets indicating the pattern of SILT under all studied conditions are shown in the Supplementary material (Figure S5). In all three mouse models, though, SILT was less abundant near the PPs (Figure 2F). To define the identity of MHC II-EGFP^+^ cells across gut-associated tissues, we analyzed their phenotype by flow cytometry in Peyer’s patches and mesenteric lymph nodes (data not shown). MHC II- EGFP^+^ cells in PP were predominantly CD19^+^ B cells and CD11c^+^ MHC II^+^ dendritic cells comprising both conventional and plasmacytoid dendritic cells, while CD11b^+^ monocytes/macrophages contributed a minor population. Similar distributions were observed in MLN. In the lamina propria and SILTs, where flow cytometry was technically limited by cell recovery, we employed confocal microscopy. Macrophages (F4/80^+^) were found only at the periphery of small SILTs and were absent in larger ones. Besides that, SILTs comprised mainly of B220^+^ B cells and CD4^+^ Th cells with the abundance of both increasing with follicle size (Figure S2-S4). These results confirm that SILTs are immunologically distinct compartments characterized by an accumulation of adaptive immune cells, particularly B cells and helper T cells. In summary, oMM12 mice exhibited the highest SILT counts but with smaller and less differentiated structures, while CV mice showed fewer but larger and functionally developed SILT units, highlighting the role of microbiota complexity in organizing and maintaining intestinal lymphoid architecture (Figure S2-S4).

#### GF and oMM12 mice show significantly smaller PP follicles than CV mice but similar counts

**Figure 3.**
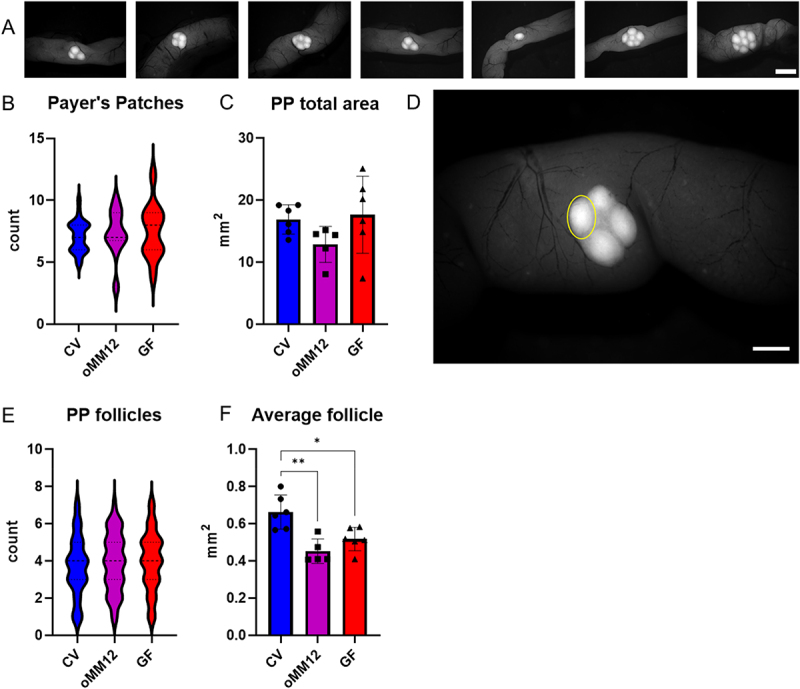
Microbiota regulate the size and morphology of mouse PPs. (A) Stereomicroscopic visualisation of individual PPs of an CV mouse ordered from left to right along the GIT direction from the duodenum to the distal ileum. Scale bar: 2 mm. (B) PP counts in the small intestine of CV, oMM12 and GF mice. (C) total area of PPs per mouse in CV, oMM12 and GF mice. *n* = 5 for GF and oMM12 mice; *n* = 6 for CV mice. (D) PP structure (CV); a subunit is indicated by a yellow circle. Scale bar: 1 mm. (E) The number of subunits in each PP of each model is graphically represented in violin plots (better capturing the variability in the distribution of values), with n(CV) = 41, n(oMM12) = 37, n(GF) = 50. (F) comparison of the average size of a PP subunit in CV, oMM12 and GF mice *n* = 5 for GF and oMM12 mice; *n* = 6 for CV mice. Significant differences were determined by ANOVA with Tukey’s multiple comparison post-hoc test and are marked with an asterisk (*p < 0.05, **p < 0.01). The bars represent the mean ± SD for each model.

Thanks to the unique morphology of PPs and their strong EGFP fluorescence signal, derived from MHC II^+^ APCs, we were easily able to determine all PPs per mouse (Figure 3A). Although PPs are large and usually show no interindividual variability in counts or overall characteristics, surprisingly PP counts varied along the length of the intestine in each mouse, but their averages were similar (Figure 3B). In CV mice, PP counts ranged from 5 to 10, averaging 7.1 ± 1.3 PPs. GF mice displayed the widest range of PP counts, from 4 to 12, albeit with a similar mean, 7.5 ± 2.2 PPs. In oMM12 mice, PP counts averaged 7.3 ± 1.9. In other words, PP counts only varied with the location in the intestine of each mouse. This distinct pattern in the longitudinal distribution of PPs is observed across all conditions (GF, oMM12, and CV mice).

As with SILT, PPs were manually counted, but a measurement tool was used to quantify additional parameters, including the PP area and discrete follicular subunits (Figure 3D). Despite some interindividual variability in the data, neither the total area of PPs (Figure 3C) nor the number of PP follicular subunits (Figure 3E) significantly varied depending on microbial colonization. However, PP follicles were significantly smaller in GF (0.51 ± 0.06 mm^2^; *p* < 0.05, one-way ANOVA with Tukey’s post-hoc test) and oMM12 (0.45 ± 0.07 mm^2^; *p* < 0.01, one-way ANOVA with Tukey’s post-hoc test) mice than in CV (0.66 ± 0.09 mm^2^) mice (Figure 3F). Based on these results, differences in the total area of all PPs mainly result from differences in the size of individual follicular subunits. Therefore, microbiota plays a key role in regulating the size and morphology of PPs, which are essential for immune priming and adaptive immune responses in the gut. In summary, while PP counts remained comparable across all mouse models, CV mice exhibited significantly larger follicular subunits than GF and oMM12 mice, underscoring the role of microbiota in shaping the structural complexity and immune competence of PPs.

### The caecal patch is dispersed into small units in GF mice but compact in CV mice, while MLN morphology remains largely unchanged

Considering these findings, we morphologically characterized immune compartments of the GIT of CV, oMM12 and GF mice. In all mice (*n* = 4–11, including the colonic lymph node – Supplementary data figure S6), no major differences in MLN morphology were observed between different mice strains (Figure S6).

**Figure 4.**
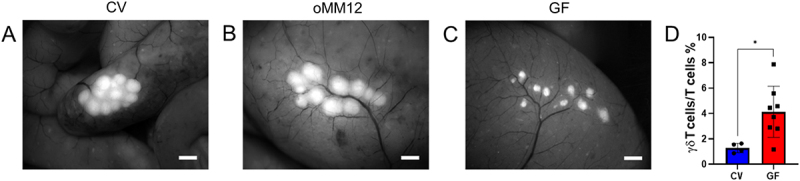
Microbiota determines the morphology of the cecal patch: stereomicroscopic visualization of the cecum in (A) CV, (B) oMM12 and (C) GF mice. Magnification 12x, scale bar: 1 mm. (D) Total γδ T cell counts of the caecal patch are significantly higher in GF than in CV mice (*n* = 8 and 4, respectively). Significant differences identified based on the unpaired t-test are marked with an asterisk (*p < 0.05). The bars represent the mean ± SD for each model.

The cecal patch displayed the most striking morphological differences between CV and GF mice. In CV mice, the cecal patch typically appeared as a single bulky unit, albeit sometimes split into two patches close to each other (Figure 4A), resembling the PPs in the small intestine. Conversely, in GF mice, the cecal patch lacked a compact structure and was dispersed into several small units throughout the enlarged cecum (Figure 4C). In oMM12 mice (Figure 4B), cecal patch was arranged as in CV mice but without a completely compact patch. In addition to these morphological differences, cecal patches in GF mice also significantly differ in the percentage of γδ T lymphocytes (*p* < 0.05, unpaired t-test) (Figure 4D). In summary, while MLN morphology remained consistent across all microbial conditions in mice, the cecal patch showed pronounced structural disruption in GF mice, indicating that microbiota is essential for its compact organization.

#### pDC counts in PPs are significantly lower in GF mice than in CV mice

After a precise and quantitative dissection of all GIT-associated secondary lymphoid organ units (PPs and MLNs), we performed flow cytometric analysis by applying two multicolor antibody panels in parallel (myeloid and lymphoid) to individual or pooled PPs and MLNs. Through this approach, we were able to comprehensively analyze immune cell populations in immune compartments of both CV and GF mice. We selected the first (duodenum and proximal jejunum), the last (distal ileum), and intermediate (jejunum and proximal ileum) PPs to account for the high variability in the number of PPs in individual mice.

**Figure 5.**
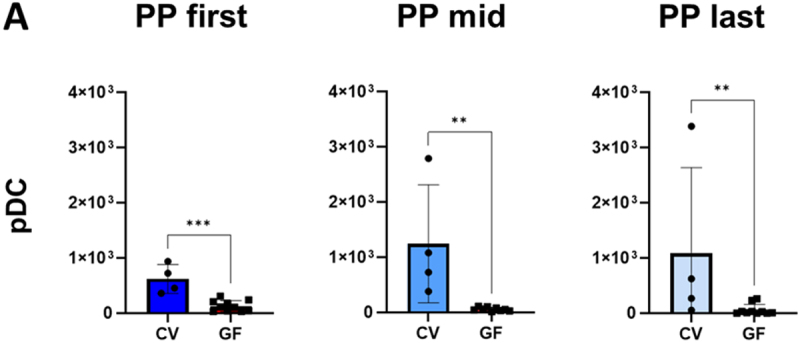
Immune cell populations in Peyer’s patches (PPs): cytometric phenotyping compares the counts of individual cell types in PP in different parts of the small intestine. PP first only includes the first PP belonging to the duodenum and proximal jejunum, PP last only includes the last PP in the ileum. PP mid comprises a variable number of PPs in between. (A) compares the total number of plasmacytoid dendritic cells (pDCs), namely CD19^−^, MHC II^+^ and BST2^+^ cells, based on myeloid panel staining. Significant differences identified based on the unpaired t-test are marked with an asterisk (**p < 0.01, ***p < 0.001). The bars represent the mean ± SD for each model.

The myeloid panel staining revealed that plasmacytoid dendritic cells (pDC) counts were significantly reduced throughout the intestine of GF mice compared to CV mice (Figure 5A) (*p* < 0.01 for the middle and last PPs, and *p* < 0.001 for the first PP, unpaired t-test). In summary, GF mice exhibited a significant reduction in pDCs in PPs compared to CV mice, with a pronounced gradient along the intestine, underscoring the essential role of microbiota in maintaining balanced immune cell populations within gut-associated lymphoid tissues.

GF mice displayed a gradual decrease in immune cell counts from the start to the end of small intestine PPs. By contrast, in CV mice, immune cell counts remained relatively unchanged throughout the small intestine (Figure S7). Some subpopulations displayed greater variability among individual mice, suggesting potentially significant changes with a larger sample.

For datasets with non-normal distribution, Kruskal–Wallis testing was applied. In GF mice, significant gradients were detected in PP. CD45^+^ cells, live cells, and B cells were significantly more abundant in the first compared to the last PP (*p* < 0.05 for all). Total T cells were significantly increased in the first compared to the mid (*p* < 0.05) and to the last PP (*p* < 0.01). This pattern was also observed for CD4^+^ and CD8^+^ T cells (*p* < 0.05 and *p* < 0.01, respectively). γδ T cells showed a significantly higher number in the first compared to the last PP (*p* < 0.05), as did CD8^+^ NKT cells (*p* < 0.05).

#### Microbiota affects the cellular composition of MLNs

Because MLNs from GF and CV mice showed no morphological difference (S6), we phenotyped and quantified relevant cell populations by flow cytometry. To assess whether MLN immune populations reflected the GIT microbiome gradient, we categorized them based on their order of drainage from the small intestine to colon. As with PPs, we also analyzed separately first and last unit and pooled intermediate (mid) ones for comparative analysis given their high variability in number in individual mice, indicating striking difference in overall live cell counts between GF and CV mice for last MLN draining distal GIT.

**Figure 6.**
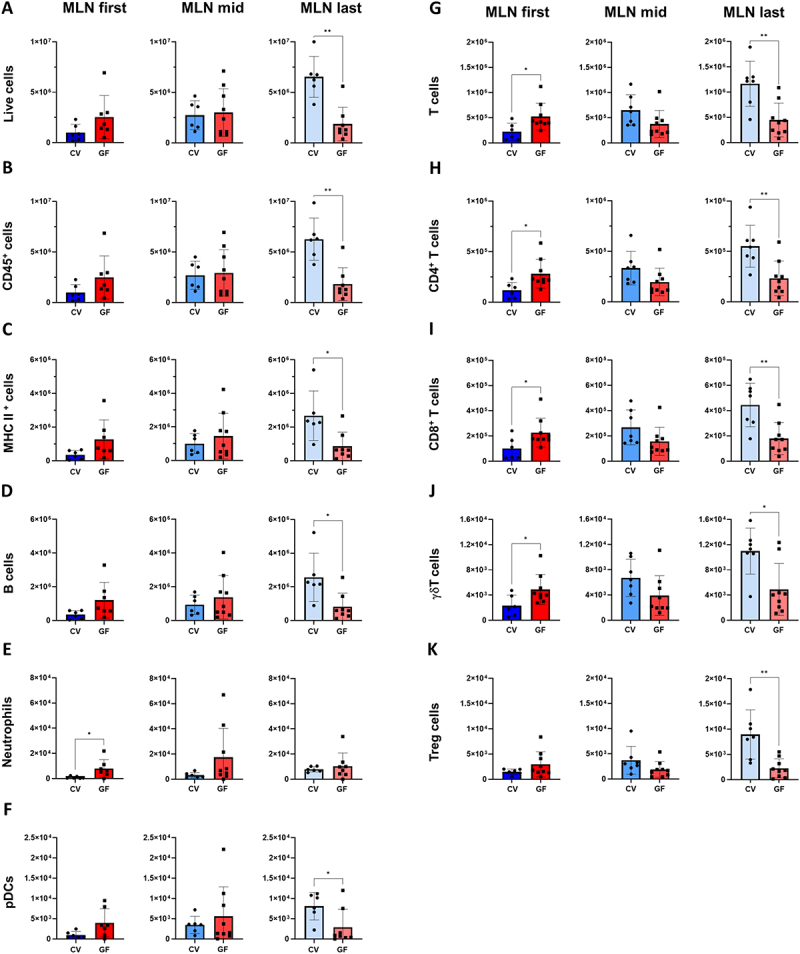
Microbiota alter immune cell populations in MLNs: detailed cytometric phenotyping of immune cell populations in different sections of the MLN complex in CV (blue) and GF (red) mice. MLN first refers to the first mesenteric node draining the proximal GIT; MLN last, to the last node in the complex that drains the colon; and MLN mid, to all nodes in between. Data were collected using a myeloid staining panel for A-F and a lymphoid staining panel for G-K. (A) Shows the number of all live cells in each segment. (B) Compares CD45^+^ populations and (C) shows MHC II^+^ cells. (D) compares B cells based on the CD19 marker. (E) Displays neutrophils defined as CD45^+^, CD19- CD11B^+^ Ly6G^+^ cells. (F) Compares plasmacytoid dendritic cells (pDCs) (CD19-, MHC II^+^ and BST2^+^ cells). (G) Shows differences in the numbers of T cells identified using the CD3 marker, which were further subdivided into (H) CD4^+^ and (I) CD8^+^ and (J) γδ T cells, and (K) the CD25 marker, primarily T regulatory cells. Significant differences identified based on the unpaired t-test are marked with an asterisk (*p < 0.05, **p < 0.01). The bars represent the mean ± SD for each model.

Specific immune cell subpopulations showed variability in MLNs, particularly in those draining the colon (the last MLN). All live cells, leukocytes (CD45^+^), APCs (MHC II^+^) and B (CD19^+^) and DCs (CD45^+^ MHC II^+^ CD19- Bst-2^+^) cells were significantly lower in GF mice than in CV mice (Figure 6A–D,F). In contrast, neutrophil counts significantly differed only in the first MLN draining the proximal GIT, with a higher number of cells in GF mice (Figure 6E). Furthermore, T (CD3^+^) cells, including both CD8^+^ and CD4^+^ subpopulations, as well as γδ T cells were significantly increased in the first lymph node of GF mice and together with CD4^+^ CD25^+^ T cells in the last lymph node of CV mice (Figure 6G–K). Statistical significance was evaluated using either the unpaired t-test or the non-parametric Mann – Whitney test, depending on the distribution of each dataset. The Mann – Whitney test was applied to the following comparisons: live cells in MLN last (*p* < 0.01); CD45^+^ in MLN last (*p* < 0.01); MHC II^+^ in MLN last (*p* < 0.05); B cells in MLN last (*p* < 0.05); neutrophils in MLN first (*p* < 0.05); T cells in MLN first (*p* < 0.05); CD8^+^ T cells in MLN first (*p* < 0.05); γδT cells in MLN first (*p* < 0.05). The unpaired t-test was used for the following comparisons: pDCs in MLN last (*p* < 0.05); T cells in MLN last (*p* < 0.01); CD4^+^ T cells in MLN first (*p* < 0.05) and last (*p* < 0.01); CD8^+^ T cells in MLN last (*p* < 0.01); γδ T cells in MLN last (*p* < 0.05); and Tregs cells in MLN last (*p* < 0.01).

**Figure 7.**
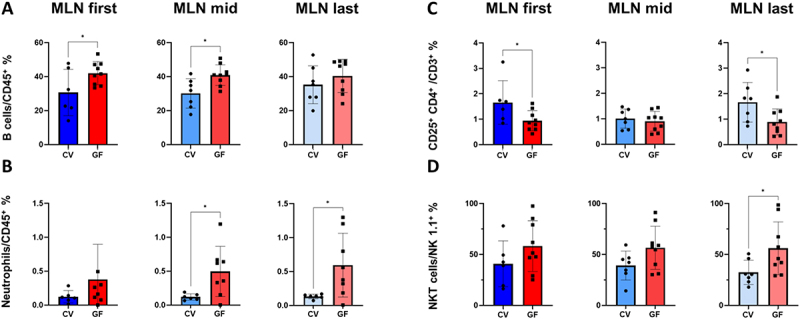
Immune cell populations in MLN (percentages): detailed cytometric phenotyping compares percentages of selected immune populations in the different sections of the MLN complex in CV (blue) and GF (red) mouse model. MLN first means the first mesenteric node draining the proximal part of the GIT, and MLN last refers to the last node in the complex that drains the colon. MLN mid includes the variable number of all nodes in between first and last. (A) Shows the proportion of B cells among all leukocytes, i.e. CD45^+^ cells. (B) Shows the percentage of neutrophils in the same population. Parts (A) and (B) show the results of myeloid panel staining. (C) shows the proportion of CD25^+^ CD4^+^ T cells among all T cells and (D) shows the proportion of NKT cells among all NK1.1 positive cells, both measured by the lymphoid panel. Significant differences identified based on the unpaired t-test are marked with an asterisk (*p < 0.05). The bars represent the mean ± SD for each model.

Along the entire length of the MLN complex, B cells (Figure 7A) and neutrophils (Figure 7B) accounted for a lower percentage of CD45^+^ cells in CV mice than in GF mice, albeit in case of last MLN for B cells and first for neutrophils non significantly. Conversely, CD25^+^ T cells (Figure 7C) accounted for a lower percentage of T cells in GF mice, but these mice also showed an increased representation of NKT cells among NK1.1 cells (Figure 7D). Moreover, cellular gradients among MLNs draining different sections of the intestine and colon were absent from GF mice, whereas CV mice showed increasing differences in most populations (Figure S8), highlighting disparities in the cell composition of MLNs draining the colon between CV and GF mice. Statistical comparisons in this analysis were performed using the unpaired t-test. Significant differences between CV and GF mice were observed in several lymphocyte subpopulations. Specifically, GF mice displayed a significantly higher proportion of B cells among CD45^+^ cells in both the first and mid MLN (*p* < 0.05 for both). Neutrophils (as a proportion of CD45^+^ cells) were also significantly more abundant in GF mice in the mid and last MLN (*p* < 0.05 for both). Additionally, CD25^+^ CD4^+^ T cells (as a proportion of CD3^+^ T cells) were significantly increased in CV mice in first and last MLN compartments (*p* < 0.05 for all). A significant increase in the proportion of NKT cells (defined as NK1.1^+^ TCRβ^+^) was also observed in the last MLN of GF mice (*p* < 0.05).

For datasets with normal distribution, one-way ANOVA with post-hoc analysis revealed several significant gradients in CV mice. CD45^+^ cells increased significantly between mid and last MLN (*p* < 0.01), and between first and last MLN (*p* < 0.0001). B cells were significantly more abundant in the last compared to the first (*p* < 0.01) and mid MLN (*p* < 0.05). pDCs showed markedly higher numbers in last MLNs relative to the first (*p* < 0.001) and mid (*p* < 0.05). Conventional dendritic cells (cDCs) increased in the end compared to both the first (*p* < 0.01) and mid MLNs (*p* < 0.05). Total T cells and were significantly elevated between mid and last (*p* < 0.05) and between first and last MLNs (*p* < 0.001). CD4^+^ T cells showed significant increase between first and last MLNs (*p* < 0.001). CD8^+^ T cells numbers are significantly increased between first and last MLNs (*p* < 0.01), γδ T cells showed a significantly increased number in the last compared to the first (*p* < 0.001), as well as a significant increase in the mid compared to the first (*p* < 0.05), and in the last compared to the mid (*p* < 0.05) and Treg cells showed a strong upward gradient (first vs. last MLN: *p* < 0.001). NKT cells also increased significantly between first and last MLNs (*p* < 0.05).

For datasets with non-normal distribution, Kruskal – Wallis testing was applied. In CV mice, neutrophils showed a significant increase between the first and last MLN (*p* < 0.01).

Combined, the findings presented above demonstrate that microbiota markedly affect immune cell populations and lymphoid organs in the GIT. Although overall cell counts did not significantly differ, specific subpopulations, such as pDCs, neutrophils, B cells, T cells and many of their subpopulations did so. Additionally, the percentage distribution of some cell populations differed between CV and GF mice, corroborating the assertion that microbiota affect the immune cell landscape in MLNs. In summary, although MLN morphology remained unchanged, GF mice exhibited marked alterations in immune cell composition compared to CV mice, including reduced counts of several APC populations, disrupted cellular gradients, and shifts in T cell subpopulations, highlighting the microbiota’s critical role in shaping the immune landscape of MLNs.

### Cornea

The GIT houses the most abundant and diverse commensal ecosystem in mice. To assess whether intestinal microbiota specifically drive dynamic structural changes in gut-associated lymphoid tissue, we compared the GIT with the cornea, a tissue that interacts with the microbiome but is distant from intestinal influence. As a fully translucent structure of the visual pathway, the cornea directly interacts with the environmental microbiota and, as such, is suitable tissue for *in vivo* observation of MHC II^+^ cells by fluorescence stereomicroscopy. Transparent cornea makes it possible to differentiate corneal APC populations such as Langerhans cell (LC)-like and macrophage (MΦ)-like cells (Figure S11). Moreover, corneal translucency enables whole eyeball observation by LSFM without additional clearing, preventing various artifacts.

Given that the corneal epithelium is exposed to environmental microbiota, we counted MHC II^+^ cells from the limbus border to the center of the eye in CV and GF mice, recording a significantly higher number of MΦ-like cells (Figure S11B) per mm^2^ of cornea in CV (36,7 ± 7,9) compared to GF (26 ± 2,3) mice (*p* < 0,001) based on the unpaired t-test. However, we observed a higher variability in APC counts among CV animals, possibly reflecting the individual microbiome status of each animal. In contrast, germ-free mice showed highly homogeneous APC counts of both LC- and MΦ-like cells.

## Discussion

Our quantitative findings showed that intestinal microbiota modulate immune cell populations and drive dynamic structural changes in GALT. Case in point, GF mice showed a significantly larger intestinal surface area than CV and oMM12-colonized mice (Figure 1A) but a lower density of villi in the distal ileum (Figure 1F). The larger intestinal surface and observed differences in SILT and PP structures in GF mice may be a compensatory mechanism for the reduced villi density, suggesting that microbiota play a crucial role in regulating intestinal morphology and function. However, the larger intestinal surface area of GF mice only partly compensated for the lower density of villi, as they contain fewer APCs than CV mice (Figure 1K). GF mice displayed a significantly smaller SILT count, area and volume (Figure 2B–E). PP follicles were significantly smaller in GF (*p* < 0.05) and oMM12 (*p* < 0.01) mice compared to CV mice, indicating that microbiota are essential for proper PP development (Figure 3F). The cecal patch was dispersed into small units in GF mice but compact in CV mice (Figure 4A–C). pDCs counts of PPs were significantly lower in GF mice than in CV mice (Figure 5). Therefore, microbiota regulate (i) SILT differentiation, (ii) PP size and morphology, (iii) cecal patch morphology and (iv) MLN cellular composition. GF mice showed profound changes in immune cell composition compared to CV mice, including reduced APC subsets, disrupted cellular gradients, and altered T cell profiles. The observed differences in SILT and PP structures suggest that microbiota may influence the development of these lymphoid tissues through cytokine signaling or direct interactions with immune cells.

The use of MHC II-EGFP knock-in mice enabled us to visualize MHC II-expressing cells across GALT compartments. Importantly, by combining flow cytometry and confocal microscopy, we could identify tissue-specific differences in MHC II^+^ cell composition. In SILTs, we provide the first detailed microscopy evidence that these structures are rich in B and CD4^+^ T cells, but largely devoid of macrophages. These data underscore the role of SILTs as organized inductive sites and highlight their immunological distinction from the lamina propria, which harbors macrophages as the dominant MHC II^+^ cell population. A limitation of the approach was the presence of autofluorescence in the green channel, which was addressed by careful co-staining and appropriate controls. Furthermore, due to the low yield of live cells from individual SILTs, flow cytometric characterization was not feasible in this compartment. Nonetheless, the combined imaging and cytometric analysis provides a robust characterization of the cellular landscape.

Microbiota affect the overall development of the intestinal immune system, as shown by differences in the abundance of specific immune cell populations between GF and CV mice, namely reduced B and T cell numbers in the small intestine, decreased colonic Th cells, and lower levels of memory and effector T cells, Tregs, and myeloid cells in GF mice.^3,7^ Our results confirm the previously reported reduction in T lymphocytes in MLNs,^3^ including both CD4^+^ and CD8^+^ T lymphocytes. However, our study reveals an additional layer of complexity, that is, this reduction is uneven along the intestine. More specifically, the largest and most distal segment of the MLN complex is primarily responsible for the overall decrease, while the pattern is reversed in the first MLN, which showed a higher number of T cells (both CD4^+^ and CD8^+^) in the GF model. Considering that the first MLN drains the proximal section of the small intestine and the last MLN drains the colon,^30^ the decreased T cells of GF mice may be related to the absence of microbial stimuli in areas where they are most abundant in the CV model. Contrary to earlier literature on BALB/c mice,^31^ though, we did not observe significant differences in either the total numbers or the relative abundance of T cells in PPs in C57BL/6 mice. The observed, differences between the two strains may be attributed to variations in their immune response profiles, particularly T lymphocytes. BALB/c mice are known to display a pronounced polarization toward a Th2 response, whereas C57BL/6 mice show a tendency toward a Th1 response.^32^

GF and oMM12 significantly differed from CV mice. In GF mice, as well as after antibiotic use, microbiota elimination markedly changes the size of intestinal segments, particularly the cecum, which is typically the richest segment in intestinal microbiota under physiological conditions.^33–35^ In turn, colonization with oMM12 essentially normalizes intestinal morphological characteristics (outer surface area, villus density in the distal ileum) but not the abundance of APCs in the lamina propria, based on the number of MHC II^+^ cells in individual villus populations (proximal jejunum vs. distal ileum). In this study, cell counts in the villi did not significantly differ between oMM12 and GF, reaching approximately half (proximal jejunum) and one-third (distal ileum) of the counts recorded in CV mice. These data indicate that the selected strains present in oMM12 are unable to normalize the immune status of the intestinal mucosa to physiological APC counts. As expected, the differences from CV mice were even more pronounced in the distal ileum because this the region of the small intestine with the most diverse microbiome composition and, accordingly, the strongest interactions with the surrounding GALT.

In both GF and oMM12 mice, a significant portion of MHC II molecules was localized in intracellular vesicular compartments. The phenotype of these cells with reduced activation status corroborates findings previously reported in the literature,^36–38^ according to which MHC II surface expression on macrophages is lower in GF than in CV mice, especially in the large intestine.

However, the large intestine was not included in our study due to previously identified limitations in the quantitative analysis of this tissue.^26^ The proximal and distal sections of the small intestine express markedly different functional and immune characteristics as a function of microbial properties, so their comparison suggests a gradient, particularly in the distal section, possibly due to the higher abundance and diversity of the microbiota. Thus, this gradient likely results from the impact of gnotobiotic states on the functional arrangement of the intestine.

The MHC II-EGFP mouse model may be suitable for assessing combinations of bacterial strains for replacing complex (yet locally specific) microbiota toward standardizing their physiological interactions with the host. However, the lack of knowledge about molecular patterns/commensal mechanisms of interaction with the host immune system limits our ability to select bacterial strains to induce a specific microbiome. In conventionally colonized individuals, complex effects on the host likely derive from the cumulative effect of individual factors from all bacterial strains, weighted by their respective abundances. Such equilibrium is absent in oMM12 mice. Using this model as an alternative to CV requires introducing modifications to the bacterial composition of oMM12, such as *L. plantarum* monocolonization, for its systemic normalization effects on the development of juvenile mice into adulthood.^19,20^ Morphological and ontogenetic differences in the immune system between oMM12-colonized and CV mice surpassed their functional differences. This result contradicts previously published findings according to which SILT maturation is independent of microbiota,^39^ but the study was conducted exclusively in the colon.

Quantitative characterization of SILT revealed significant differences between individual microbiome models, indicating a volumetric gradient. These findings do not align with previously published data on the ileum (through intestinal tissue sectioning),^40^ with significantly fewer units than those observed in our study and a fewer ILFs in GF mice than in CV mice, in contrast to nonsignificant differences in our data (Figure 2B). Nevertheless, our findings corroborate the results from a previous study on the C57BL/6 mouse strain, which examined the entire small intestine and did not find any significant difference in SILT counts either.^39^ In line with this are also other studies conducted with the BALB/c mouse strain also reported similar CP counts in GF and CV mice^41^ and nonsignificant differences in PPs between these two models.^13^ However, differences in the sizes of individual PP subunits have previously been described in the BALB/c mouse model.^42^ Moreover, the study reported that individual SILT units were smaller in the GF model,^13^ as in our observation of a reduced overall SILT area in these mice. GF mice had approximately half of the total SILT volume of CV mice, mirroring the ratios of APCs in the lamina propria. This finding underscores the key role of microbiota in the functional differentiation of GALT.

While CPs and ILFs together form SILT, distinguishing them clearly across different colonization states remains challenging. It is well established that ILFs develop from CPs in response to microbial cues, and that their maturation is microbiota-dependent.^43^ In our study, we did not attempt to distinguish CPs and ILFs separately but instead quantified and analyzed SILT as a unified compartment. Although stereomicroscopic quantification revealed that oMM12 mice had the highest number of SILTs, confocal imaging of representative individual units across models did not indicate marked differences in their size or maturity. The discrepancy likely results from sampling limitations, as confocal microscopy focused on areas with typically larger SILTs and only a few structures were analyzed per condition. Furthermore, multiple attempts to isolate viable SILTs for flow cytometric analysis failed due to their fragility and the associated high mortality of immune cells during dissection. Thus, although our data demonstrate microbiota-dependent variation in SILT abundance, further studies are needed to fully resolve CP-to-ILF progression and its immunological implications in different microbiota settings.

An important observation in our study is the identification of a previously undescribed structure within the small intestinal mucosa, which we have termed the immunovillus. This structure, characterized by dense APC infiltration within villous projections, was observed predominantly in gnotobiotic oMM12 mice and, to a lesser extent, in GF mice. These models are defined by their restricted microbial exposure, suggesting that the immunovillus may represent a context-dependent adaptation to a limited microbiota rather than a universal feature of the GALT. Interestingly, structures resembling lymphocyte-rich villi have been previously described in humans under the term “lymphocyte-filled villi” (LFV)^44^ In that study, T cell–dominated LFVs were associated with physiological immune responses, while B cell – dominated LFVs emerged in the context of inflammatory or pathological conditions.

While the immunovillus shares some histological resemblance to LFV, several distinctions are noteworthy. First, the immunovillus appears to be most prominent in microbiota-restricted settings and has not yet been observed in conventionally colonized mice. Second, the use of MHC II-EGFP reporter mice in our study revealed localized MHC II enrichment in these structures, pointing to active antigen presentation and immune engagement at the site. This raises the possibility that the immunovillus may serve as a functional analogue to B cell–enriched LFVs described in human pathology. However, given differences in species, microbial context, and immune architecture, the immunovillus may also represent a murine-specific or microbiota-driven adaptation with no direct human equivalent.

Further investigation is needed to determine whether the immunovillus arises transiently during specific developmental windows, whether it can be induced under defined microbial or inflammatory stimuli in conventional settings, and to what extent it parallels human lymphoid structures in function and ontogeny. Comparative studies across microbial conditions and host species will be critical to understanding the immunological significance and potential conservation of this novel structure.

We also observed significant differences in γδT cell populations in cecal patch in GF and CV mice, however, this can be the result of a contamination which cannot be ruled out even despite careful excision for cecal patches in GF mice because they do not form a compact structure as in CV mice but are instead scattered. Similarly, T lymphocytes have a higher survival rate in dissociation protocols than intestinal epithelial cells, so the proportion of γδ T cells in our samples might have been artificially inflated due to contamination from surrounding tissues.

Important aspect which may contribute to several of the observed differences – particularly in the cecal patch – is the role of germinal center (GC) formation in B cell activation. It is well established that colonization of GF mice induces GC responses in Peyer’s patches, leading to increased size and structural complexity, as shown in recent works.^45,46^ A similar process may also occur in the cecal patch, which shares architectural and immunological features with Peyer’s patches and can function as a major inductive site for immune responses. Moreover, under certain conditions, SILT can host ectopic GC-like structures. Although we did not include specific markers of proliferation or GC formation (e.g., Ki67, GL7) in our analysis, the enlarged and more structured cecal patches observed in CV mice may reflect a higher frequency of activated B cells and ongoing GC reactions in response to complex microbiota. This possibility is consistent with the overall trend of increased lymphoid maturation and B cell compartmentalization in CV conditions. Future studies will be needed to investigate the presence and localization of GC B cells and proliferative activity in these tissues using appropriate markers.

It is also interesting to note that at the level of whole gut morphology, GF mice show significantly more variability compared to CV mice, both in external gut surface area and number of PPs per individual. This suggests a distinct role for the microbiota in regulating GIT ontogeny, which is at least partially mediated by the strains selected in the oMM12 model. Our data therefore indicate that stochastic morphogenetic events, combined with epigenetic and antigenic factors and cognitive experience, could introduce significant variability among mice with virtually identical genotypes. This undermines the reproducibility of experimental mouse models, particularly under non-physiological conditions. Our findings underline the need for caution among researchers conducting microbiologically controlled experiments across laboratories aimed at enhancing reproducibility in animal studies. It is critical to interpret data carefully in relation to the individual composition of lymphoid organs. The hidden morphogenic plasticity of lymphoid organs observed under non-physiological microbiological conditions may resemble the phenotypic variability triggered in various models by stress responses involving HSP90 proteins, pointing to the potential molecular mechanism.^47^ To improve reproducibility in future mouse experiments, it will be essential to identify or develop mouse strains with reduced lymphoid organ phenotypic plasticity compared to the B6 mouse strain examined in this study.

From the above findings, it is clear that microbiota colonization plays a critical role in the development of the intestinal immune system, both at the morphological level and at the level of individual cell populations. This is also reflected in the distribution of MHC II within individual APCs. These differences may limit the generalization of insights gained from models with very limited microbiota, such as single colonization experiments. The oMM12 mouse model, while initially appearing to approximate the physiological state, is far from identical. This is most evident in SILT, where similar cell numbers in the compartment are achieved through morphologically distinct compensatory mechanisms. It is evident that some properties of the intestinal immune system can only be restored by full colonization with a complex microbiota. Differences in the composition and architecture of immune tissues in gnotobiological models may be greater and more complex than they initially appear. Therefore, these models must be thoroughly explored before extrapolating results to physiological conditions.

More recently, the OMM19.1 consortium has been introduced as an expanded version of the original oMM12.^24^ This community includes additional strains from the miBC collection – such as *Thomasclavelia ramosa, Adlercreutzia mucosicola, Escherichia coli (E. Coli), Extibacter muris, Flintibacter butyricus, Ligilactobacillus murinus, Mucispirillum schaedleri, Parabacteroides goldsteinii*, and *Xylanibacter rodentium*—thereby covering a broader taxonomic and functional spectrum. Notably, OMM19.1 includes species with the capacity to produce secondary bile acids and degrade dietary fiber, which are absent in oMM12. Mice colonized with OMM19.1 exhibit physiological and immunological profiles more closely resembling those of SPF animals. Therefore, testing OMM19.1 in our model could help determine whether these additional strains possess the capacity to restore a more complete or “normalized” intestinal immune landscape.

In parallel, we have also validated another simplified microbiota – GM15—composed of 15 strains from seven of the twenty most prevalent bacterial families found in the fecal microbiota of C57BL/6J SPF or SOPF mice. GM15 was designed to reflect the functional capacity of the SOPF metagenome and yields animals that are phenotypically similar to SOPF mice across different facilities. Importantly, both GM15 and OMM19.1 contain *E. coli*, a bacterium notably absent from oMM12, suggesting that *E. coli* may contribute meaningfully to shaping intestinal immune responses.

Moreover, the addition of *Candidatus arthromitus* (commonly referred to as segmented filamentous bacteria, SFB) to synthetic communities has been shown to potently induce Th17 responses, with well-characterized downstream effects on mucosal immunity.^48^ The integration of such taxa into synthetic consortia may therefore be critical for achieving a more complete immune maturation.

Together, these findings support the hypothesis that taxa such as *E. coli* and SFB, along with other members of the OMM19.1 or GM15 consortia, may serve as candidates for augmenting the oMM12 microbiota in order to reconstitute a more physiologically representative immune phenotype.

Another important factor influencing immune system development in gnotobiotic animals is the timing of microbial colonization. Several studies have demonstrated that immune imprinting is tightly linked to the age at which colonization occurs. For instance, Hansen et al.^49^ showed that colonization of GF mice at three weeks of age – unlike colonization at one week – resulted in permanent changes to gut microbiota composition and was associated with a marked increase in pro-inflammatory immune responses. Furthermore, the same study reported that a delayed colonization window led to lasting alterations in systemic immune cell populations, including regulatory T cells, NK and NKT cells, and cytokine production, suggesting the existence of a critical postnatal period during which microbial exposure shapes the lifelong immune phenotype of the host.

Consistent with this, Nabhani *et al*.^50^ described a programmed “weaning reaction” in which the intestinal microbiota elicits a defined immune activation event at the time of weaning. Disruption or inhibition of this response was associated with pathological immune imprinting and an increased risk of developing colitis, allergic inflammation, and cancer later in life. Mechanistically, the beneficial imprinting required both microbial and dietary cues – specifically short-chain fatty acids and retinoic acid – which drove the development of RORγt^+^ regulatory T cells. Together, these findings underscore the importance of early-life microbial exposure and support the notion that the immunological outcomes of gnotobiotic models are not only shaped by microbial composition but also by the temporal dynamics of colonization.

While our study provides insights into the role of microbiota in intestinal immune system development, the use of oMM12 mice may limit the generalizability of our findings to more complex microbiota environments. Future studies should explore the specific mechanisms by which microbiota influence the development of intestinal lymphoid tissues and immune cell populations. Additionally, further research is needed to determine the potential therapeutic applications of these findings in conditions such as inflammatory bowel disease.

Moreover we wanted to look at other organs that are in close interaction with microbiota to determine microbiota influence outside of the GIT. The healthy ocular surface hosts stable, low-diversity microbial communities, primarily *Corynebacterium*, *Propionibacterium*, and *Staphylococcus* in humans. These microbes regulate immune responses by interacting with Toll-like receptors, promoting homeostasis and protecting against infections through immune cell activation and cytokine production.^51^ External factors like contact lenses, cleaning solutions, and preserved medications can disrupt this delicate microbial balance, altering diversity and composition.^52^ Although the cornea is anatomically distant from the GIT, both are key sites for studying mucosal immunity due to their continuous microbial interactions. The cornea, being more accessible, provides a distinct environment for investigating immune responses compared to the GIT. Corneal immunology in GF conditions, remains underexplored, as does the application of LSFM in combination with fluorescently tagged mouse models. Our findings, showing stable LC-like cell counts and reduced macrophage-like cell counts between CV and germ-free conditions, provide novel insights and advance the field.

## Conclusions

The MHC II-EGFP knock-in mouse is a highly suitable model for quantitative immunological research, particularly for assessing microbiota effects in lymphoid organ physiology. Our study aimed to investigate the impact of microbiota on the development and function of GALT structures. The results demonstrate that microbiota significantly influence the morphology and immune cell populations of SILT, PPs, and MLNs, confirming the critical role of microbiota in shaping the host immune system.

Our findings show that GF mice exhibit reduced APC counts and smaller SILT and PP structures compared to CV mice, highlighting the critical role of microbiota in the development of these lymphoid tissues.

This interplay cannot be fully replicated using colonization with simplified oMM12 bacterial consortium. The oMM12 microbiome partly normalizes intestinal morphology but fails to restore physiological APC counts and to fully replicate the functional immune status of CV mice, so a more refined selection of microbial strains is needed for a balanced interaction with the host immune system. GIT development is closely associated with microbiota-derived stimuli, with significant implications for the development and distribution of GALT structures. In response to the absence of a complex microbiota, compensatory mechanisms, such as the formation of ‘immunovilli,’ may reflect adaptive changes in the intestinal immune system in these models. Reduced microbiome models, such as oMM12, must be further refined to improve their efficacy in replicating natural microbial-host interactions. Therefore, standardizing microbiome research requires carefully analyzing microbial diversity and its impact on host physiology.

## Supplementary Material

Supplemental Material

## Data Availability

All sequencing data are available in the NCBI Sequence Read Archive (SRA) under BioProject accession number PRJNA1272323. Other data that support the findings of this study are available from the corresponding author, [Cerny J], upon reasonable request.
