# Supplementary material for: Microbiota modulate immune cell populations and drive dynamic structural changes in gut-associated lymphoid tissue
Source: Gut Microbes. 2025 Aug 13;17(1):2543908. doi: 10.1080/19490976.2025.2543908 (PMC12351735; doi:10.1080/19490976.2025.2543908)
Supplement: Supplemental Material [file KGMI_A_2543908_SM6697.docx]

**SUPPLEMENTARY DATA**

B 220

CD 4

CD 45


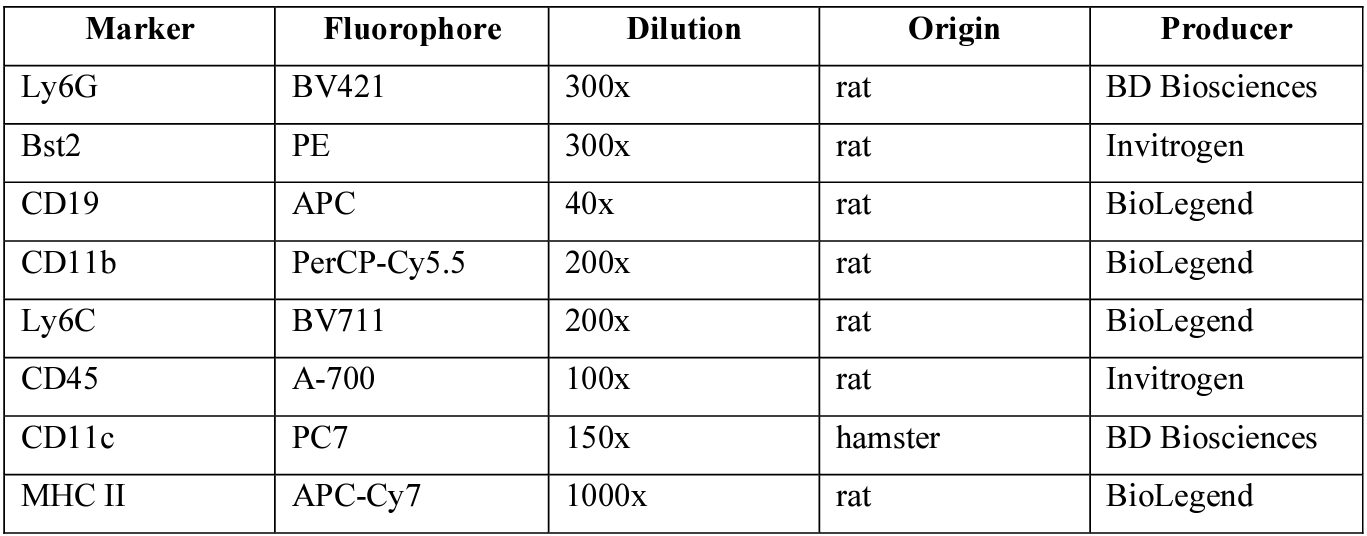


**Table I: Myeloid panel**


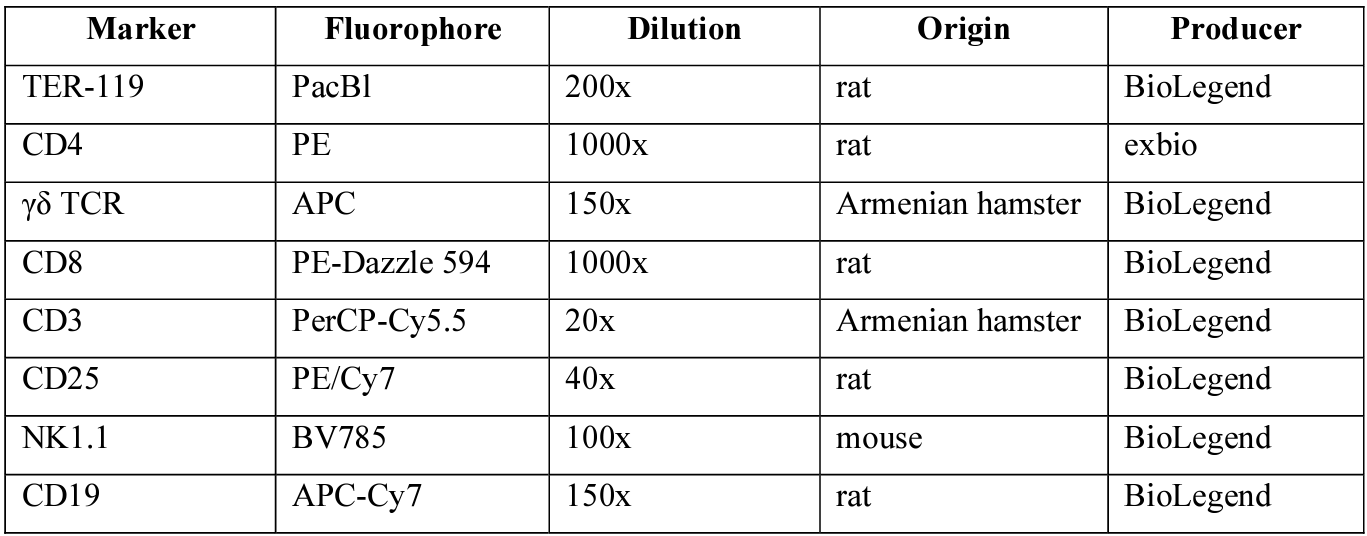


**Table II: Lymphoid panel**

**
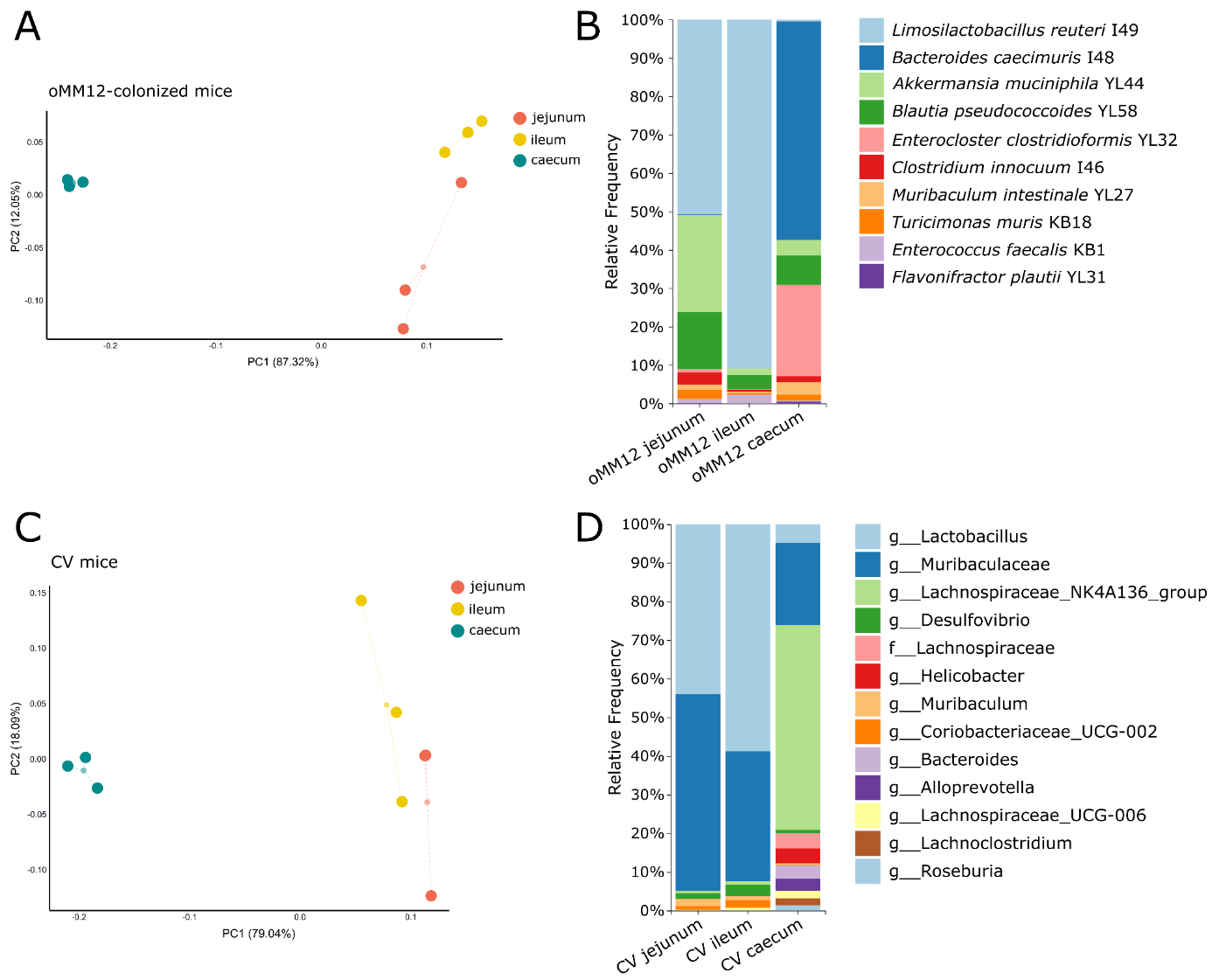
**

**Figure S1: Relative abundance of the microbiota in distinct parts of the intestine in oMM12 and conventional mice. (A) A PCA plot of the microbial communities in the jejunal, ileal and caecum of oMM12-colonized mice (n=3). Communities were plotted based on a measure of weighted UniFrac distance between the communities. (B) A chart summarizing the pooled per cent abundance of the jejunal, ileal and caecal oMM12 microbiota classification using the 16S rRNA gene. (C) A PCA plot of the microbial communities in the jejunum, ileum and caecum of conventional mice (n=3). Communities were plotted based on a measure of weighted UniFrac distance between the communities. (D) A chart summarizing the pooled per cent abundance of the jejunal, ileal and caecal conventional microbiota classification using the 16S rRNA gene.**

**
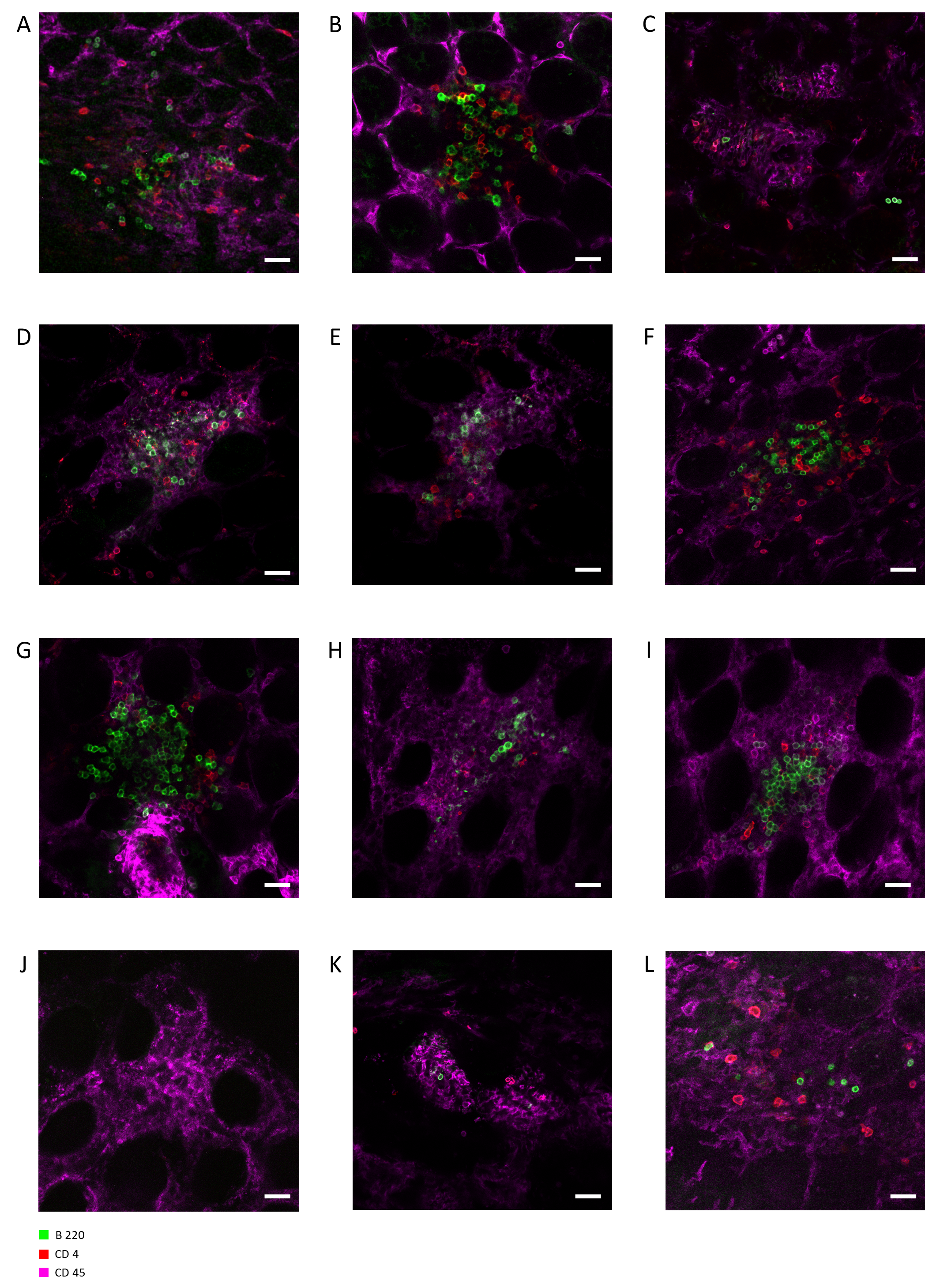
**

**Figure S2. Cellular composition of individual SILT structures in the CV mouse model.**
Panels (**A–L**) illustrate the diversity of cellular organization across solitary intestinal lymphoid tissues (SILT). CD45⁺ immune cells are shown in magenta, B220⁺ B cells in green, and CD4⁺ T helper cells in red, representing the dominant T cell subset. Images were acquired using confocal microscopy. Scale bar: 20 μm.

**
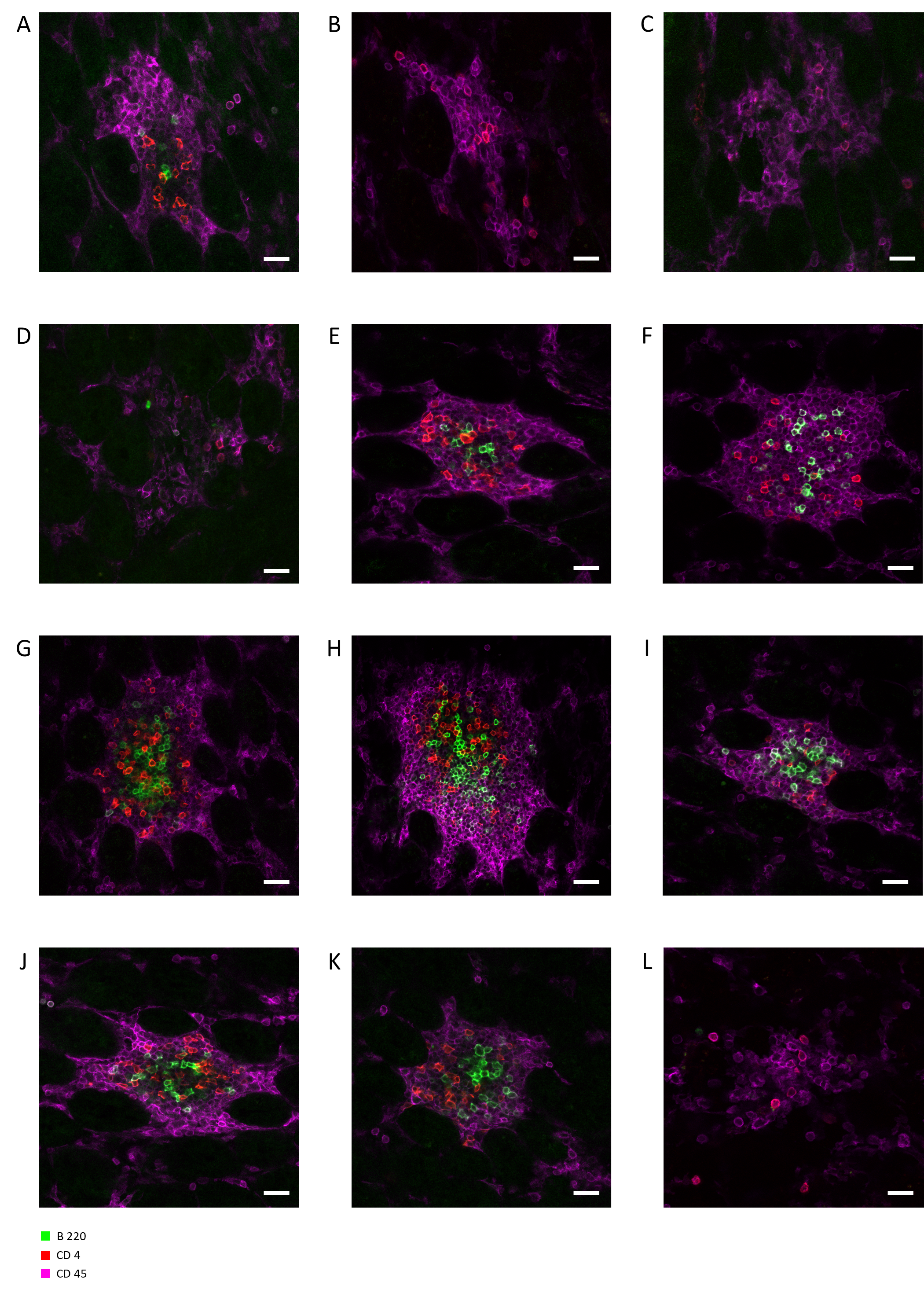
**

**Figure S3. Cellular composition of individual SILT structures in the oMM12 mouse model.**
Panels (**A–L**) illustrate the diversity of cellular organization across solitary intestinal lymphoid tissues (SILT). CD45⁺ immune cells are shown in magenta, B220⁺ B cells in green, and CD4⁺ T helper cells in red, representing the dominant T cell subset. Images were acquired using confocal microscopy. Scale bar: 20 μm.

**
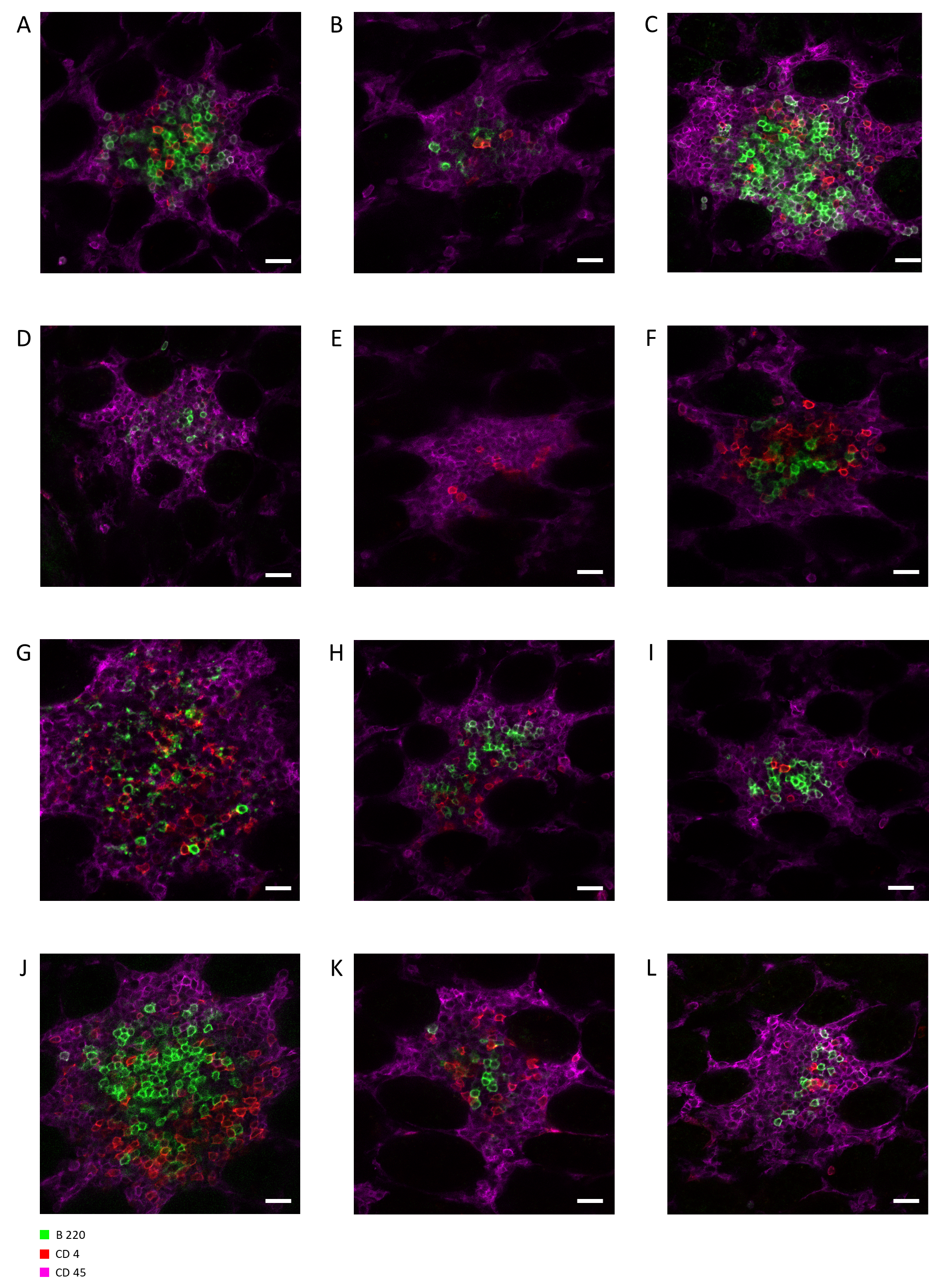
**

**Figure S4. Cellular composition of individual SILT structures in the GF mouse model.**
Panels (**A–L**) illustrate the diversity of cellular organization across solitary intestinal lymphoid tissues (SILT). CD45⁺ immune cells are shown in magenta, B220⁺ B cells in green, and CD4⁺ T helper cells in red, representing the dominant T cell subset. Images were acquired using confocal microscopy. Scale bar: 20 μm.

**
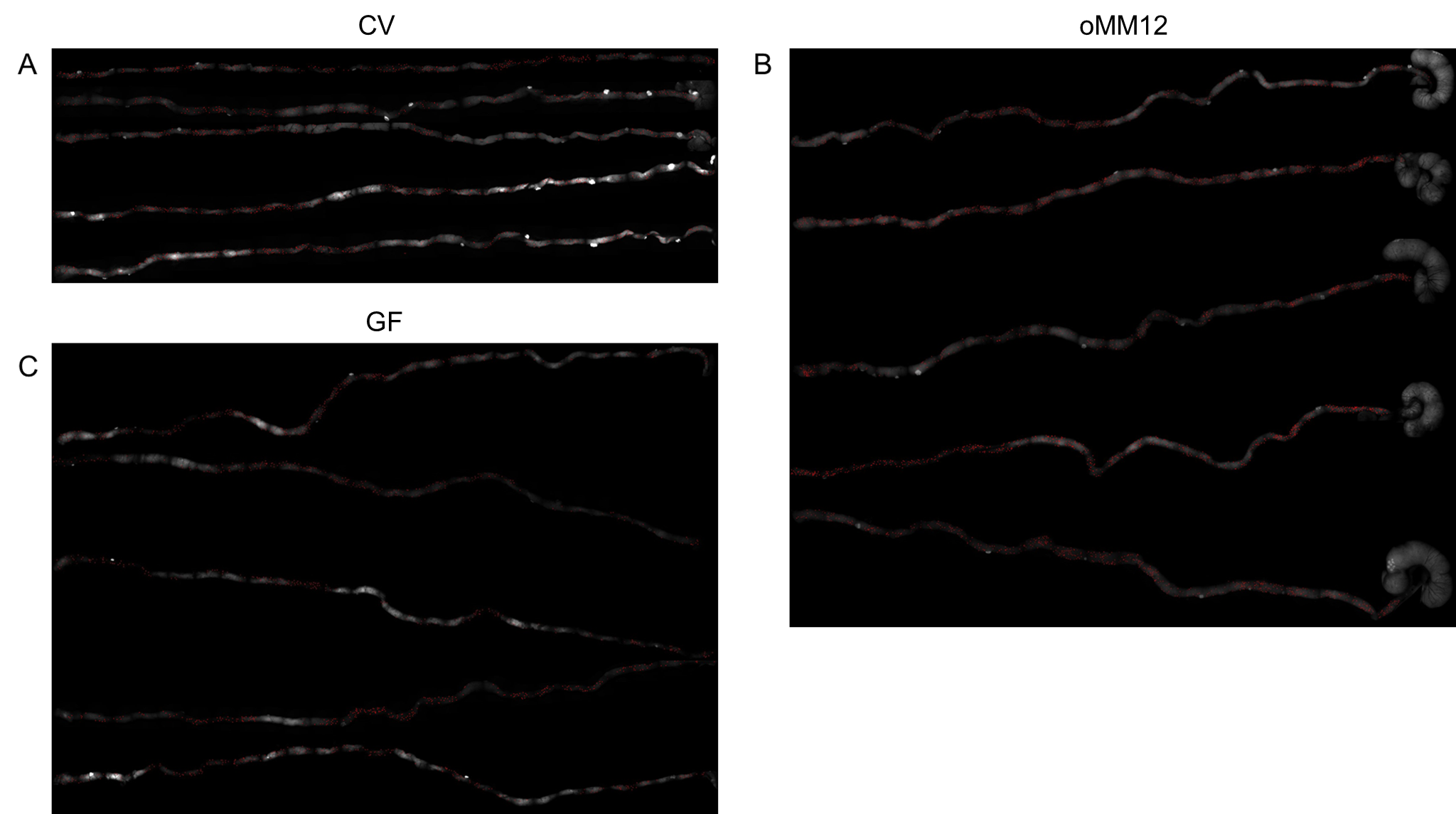
**


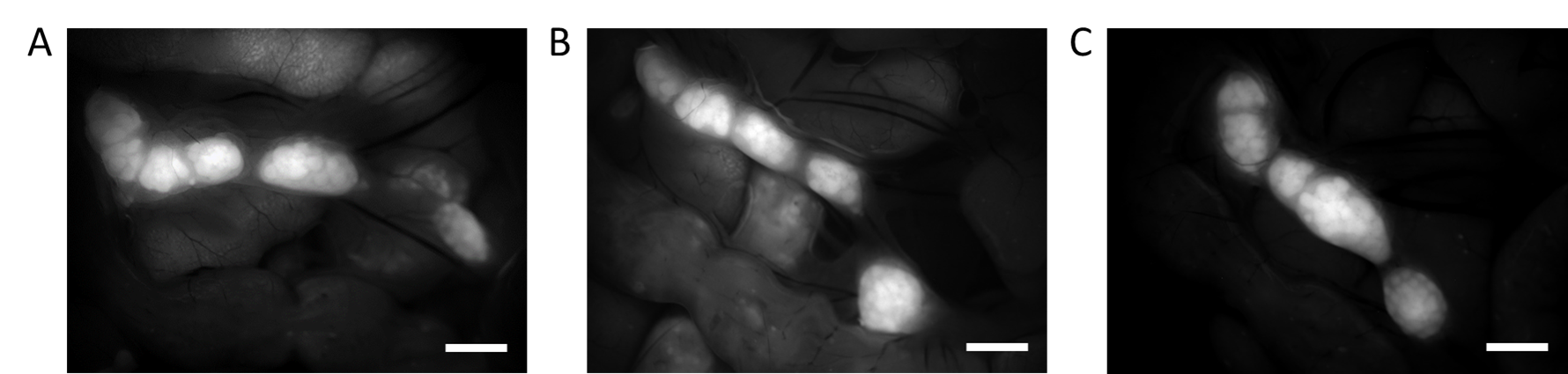


GF

oMM12

CV

**Figure S5:Visualization of entire intestines and the overall distribution of SILT in different models .** Stereomicroscopic image comparing the distribution of individual SILT follicles in the intestine of **(A)** CV, **(B)** oMM12 and **(C)** GF mice. Images of individual intestines are created by fusion of 16x magnified views.

**
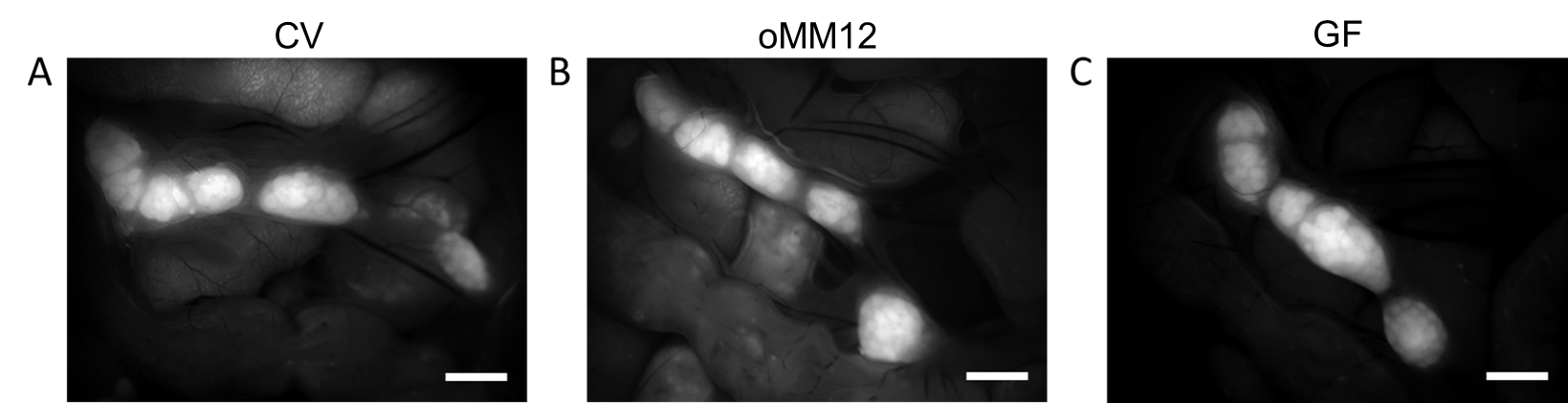
**

**Figure S6: Comparison of MLM morphology in different models.** Stereomicroscopic image comparing the morphology of MLNs in **(A)** CV, **(B)** oMM12 and **(C)** GF mice. Magnification: 9x. Scale bar: 2 mm long.

**
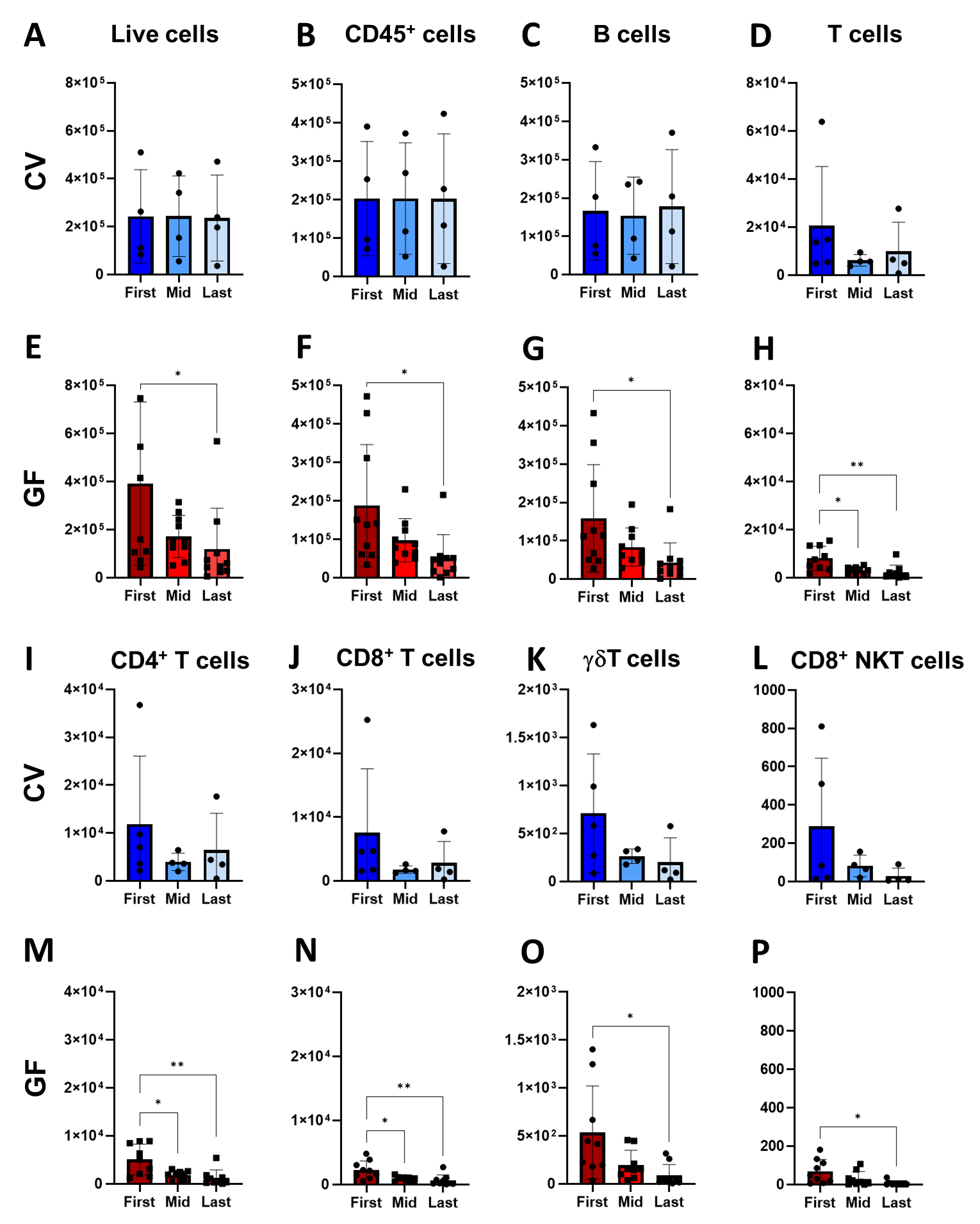
**

**Figure S7: Longitudinal gradients in the abundance of individual cell populations in PPs:** The columns in blue show the individual parts of the PPs in those of the CV model, and the red columns show the same for GF mice. (PP) **first** means the first PP in the area of duodenum/proximal jejunum, and (PP) **last** refers to the last PP in the distal ileum. (PP) **mid** includes all PPs in between first and last. **A** and **E** show the total number of live cells in the PPs from proximal to distal SI. **B** and **F** similarly show CD45^+^ cells, from which CD19^+^ (B cells) were selected in **C** and **G**. T cells in **D** and **H** were recognized by the expression of CD3. These were further subdivided by CD4 (**I** and **M**), CD8 (**J** and **N**) and γδ T receptor expression (**K** and **O**). CD8^+^ NKT cells in **L** and **P** were defined using CD3, NK1.1 and CD8 markers. Significant differences were determined by ANOVA with Tukey's multiple comparison post-hoc test and are marked with an asterisk (**p* < 0.05, ***p* < 0.01, ****p* < 0.001).


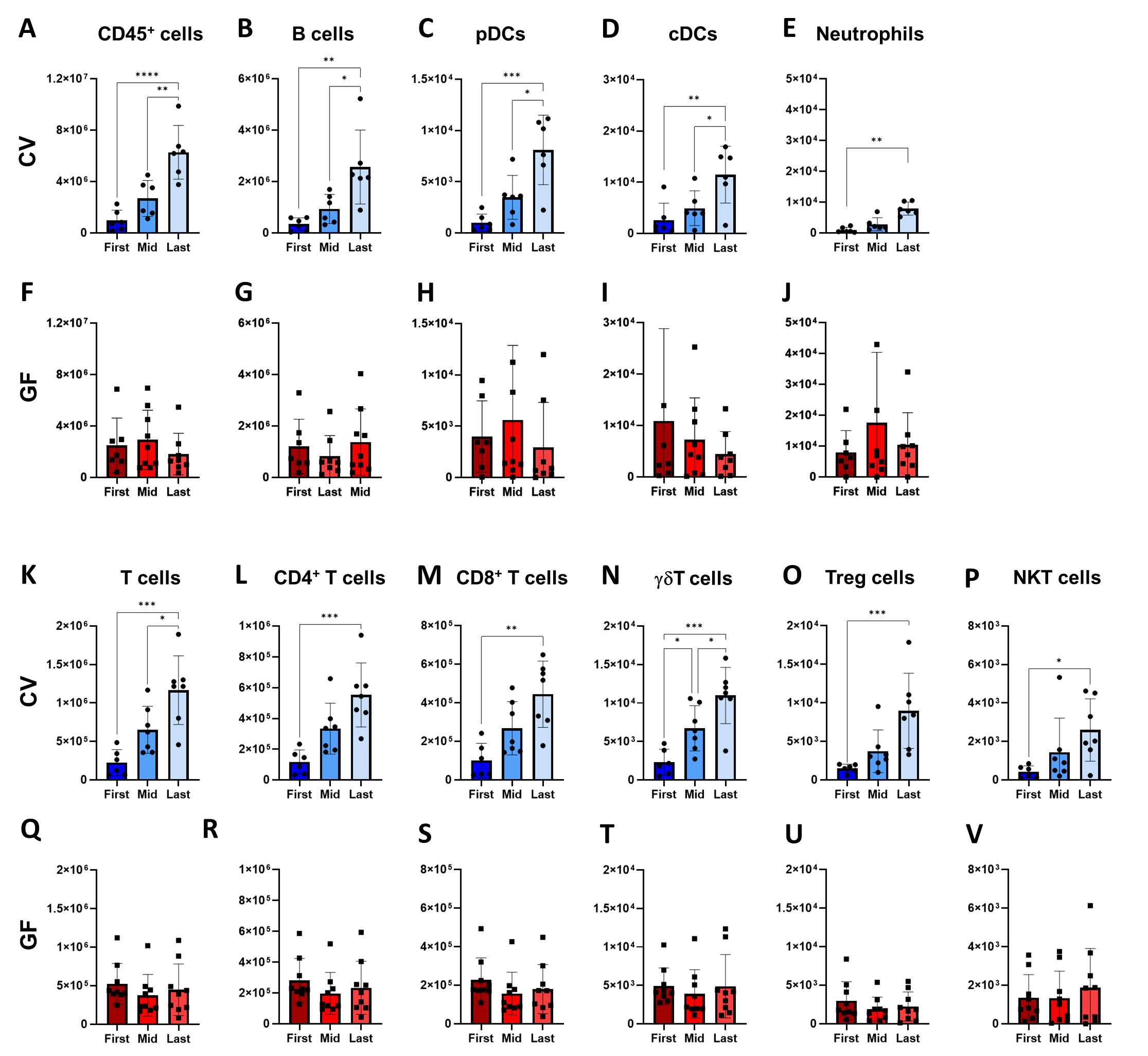


**Figure S8: Longitudinal gradients in the abundance of individual cell populations in MLN:** For the measurement of parts **A** - **J**, a myeloid panel was used. **K** - **V** were stained with the lymphoid panel. The columns in blue show the individual sections of the MLN complex in those of the CV model and the red columns show the same for the GF. (MLN) **first** means the first mesenteric node draining the proximal part of the GIT, and (MLN) **last** refers to the last node in the complex that drains the colon. (MLN) **mid** includes all nodes in between first and last. **A** and **F** show the gradient of CD45^+^ cells, i.e. leukocytes, in CV and GF mice, respectively. From these, B cells in the **B** and **G** sections were then selected by the antibody against CD19. pDCs in **C** and **H** were defined as CD19^-^, MHC II^+^, BST2^+^ cells. cDC in **D** and **I** were identified via MHC II and CD11c expression. CD11b^+^ and Ly6G^+^ cells are described as neutrophils in parts **E** and **J**. **K** and **Q** depict a gradient in T cells (CD3^+^), which are further subdivided into CD4^+^ (**L** and **R**) and CD8^+^ (**M** and **S)**. γδ T receptor distinguishes γδ T cells in **N** and **T**, and expression of CD25 determines mainly regulatory T cells in **O** and **U**. In addition to CD3^+^, NKT cells (**P** and **V**) are also defined by expression of the NK1.1 molecule. Significant differences were determined by ANOVA with Tukey's multiple comparison post-hoc test and are marked with an asterisk. (*p < 0.05, **p < 0.01, ***p < 0.001, ****p < 0.0001).


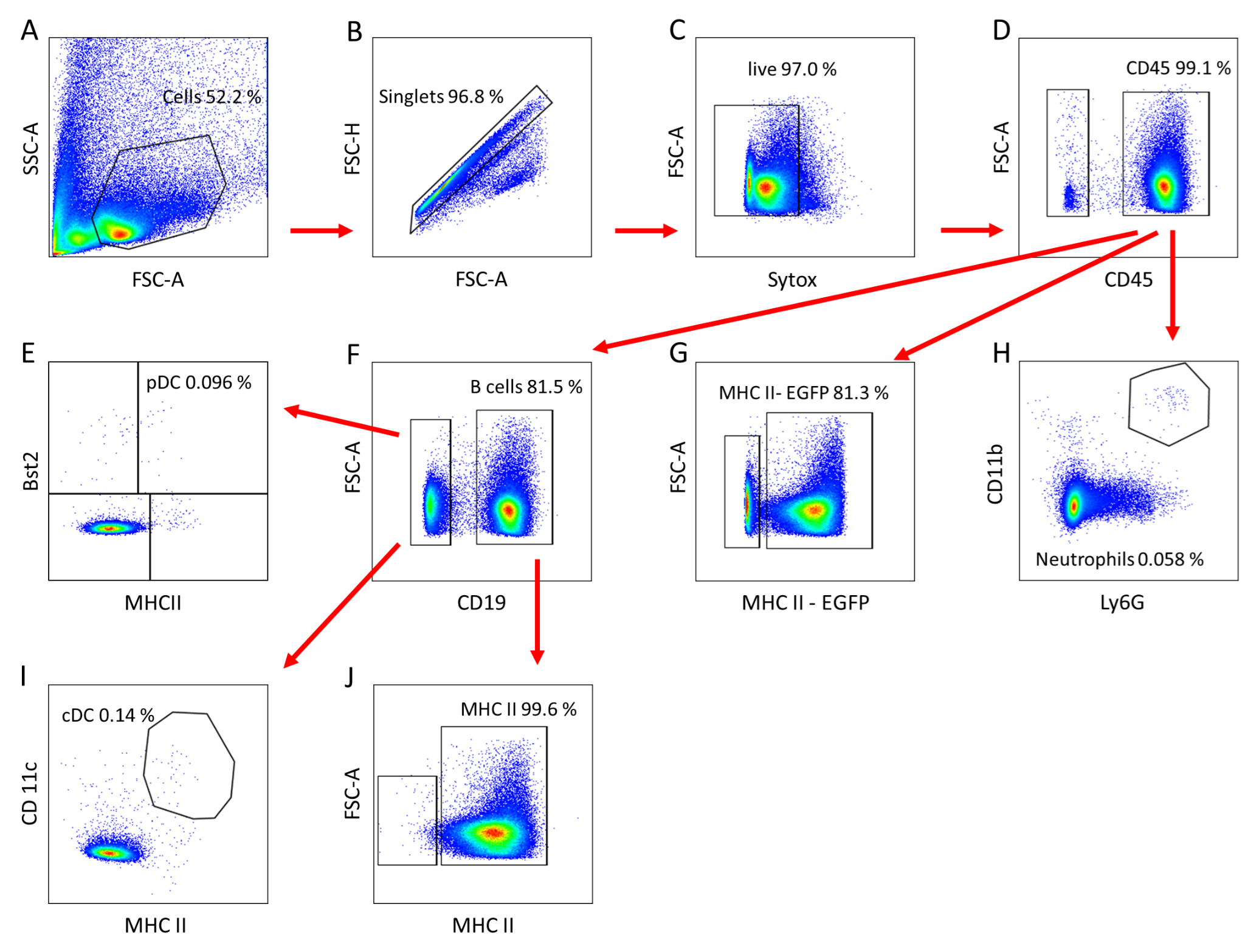


**Figure S9: Gating strategy - Myeloid panel.** Initially, cells were gated **(A)**, followed by the selection of singlets **(B)**, from which viable cells were subsequently identified **(C)**. Leukocytes were identified using the CD45 marker **(D)**, followed by the selection of CD19 negative cells **(F)** to determine pDC **(E)** and cDC **(I)** populations. CD19 positive cells were classified as B lymphocytes, with their phenotype further verified by MHC II expression **(J)**. From the leukocyte population, MHC II-EGFP positive cells **(G)** and neutrophils **(H)** were also selected.


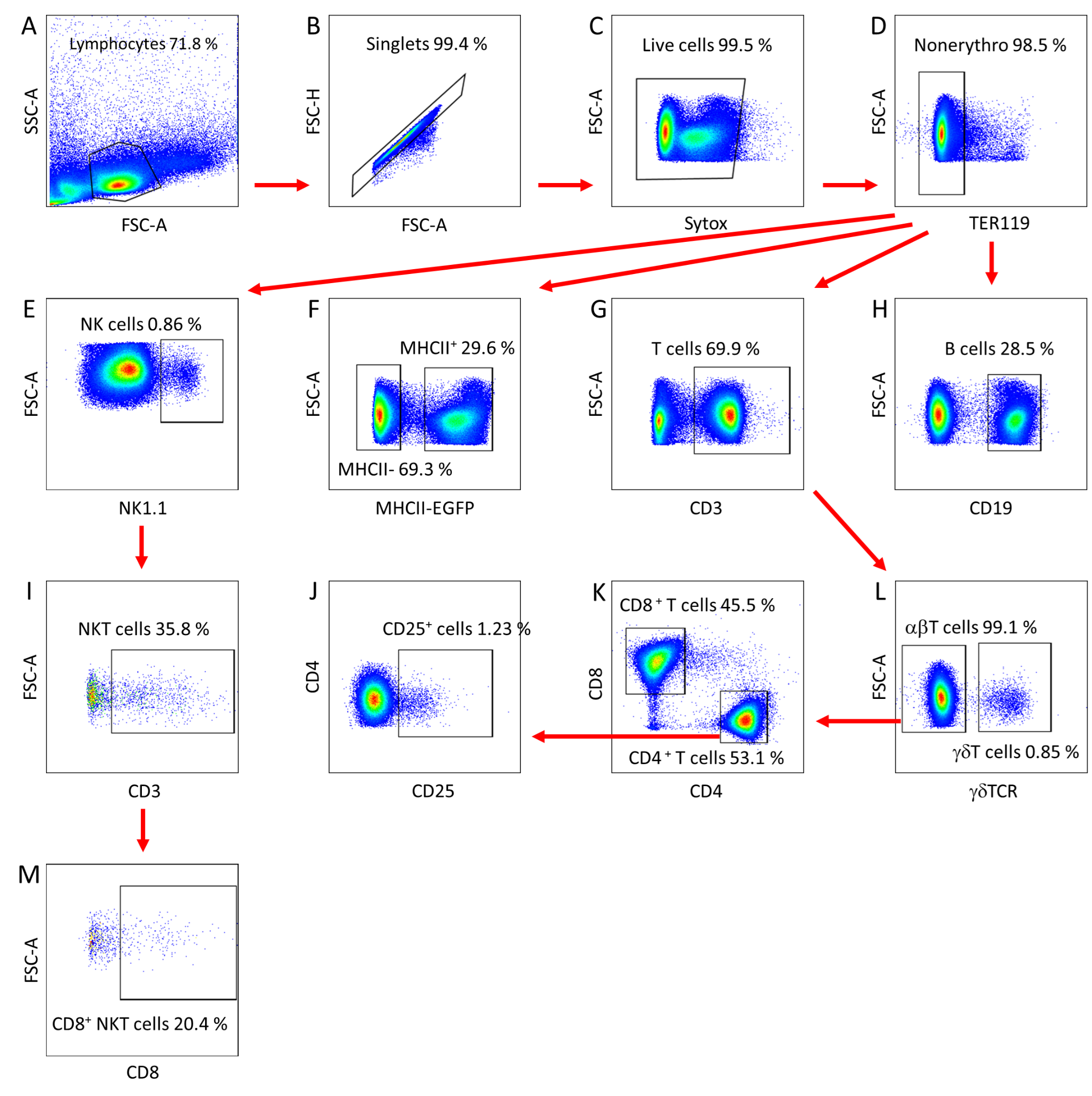


**Figure S10: Gating strategy - Lymphoid panel.** Initially, cells were gated **(A)**, followed by the selection of singlets **(B)**, from which viable cells were subsequently identified **(C)**. Then, the erythrocytes were separated **(D)** and NK1.1 positive cells **(E)** were gated from non-erythroid cells and further divided into NK cells and NKT cells **(I)**, in which the expression of CD8 molecule was determined **(M)**. The fraction of non-erythroid cells was further divided according to MHC II expression **(F)** and T **(G)** and B cells **(H)** were selected. T cells were further subdivided into αβ T cells and γδ T cells **(L)**, and αβ T cells were then fractionated into CD8 and CD4 cells **(K)**, which were further analyzed for CD25 expression **(J).**

**
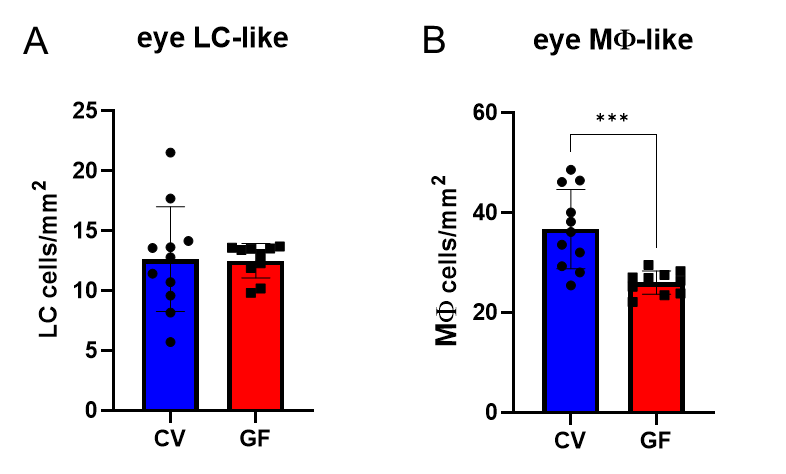
**

**Figure S11: MHC II^+^ cells in Cornea.** Comparison of **(A)** Langerhans (LC)- and **(B)** macrophage (MΦ)-like cells in CV and GF. Significant differences identified based on the unpaired t-test are marked with an asterisk (***p < 0.001), n = 11 (CV), 10 (GF).
